# Tobacco TTG2 regulates vegetative growth and seed production via the predominant role of ARF8 in cooperation with ARF17 and ARF19

**DOI:** 10.1186/s12870-016-0815-3

**Published:** 2016-06-02

**Authors:** Jun Ge, Baoyan Li, Dan Shen, Junyi Xie, Juying Long, Hansong Dong

**Affiliations:** Plant Growth and Defense Signaling Laboratory, State Ministry of Education Key Laboratory of Integrated Management of Crop Pathogens and Insect Pests, Nanjing Agricultural University, Nanjing, 210095 China; Yantai Academy of Agricultural Sciences, Yantai, 265500 China

## Abstract

**Background:**

Plant TRANSPARENT TESTA GLABRA (TTG) proteins regulate various developmental activities via the auxin signaling pathway. Recently, we elucidated the developmental role of tobacco (*Nicotiana tabacum* L.) NtTTG2 in association with 12 genes that putatively encode AUXIN RESPONSIVE FACTOR (ARF) proteins, including NtARF8, NtARF17, and NtARF19. Here we show that NtTTG2 regulates tobacco growth and development by involving the *NtARF8*, *NtARF17*, and *NtARF19* genes, with the *NtARF8* gene playing a predominant contribution.

**Results:**

Independent silencing of the *NtARF8* gene more strongly repressed tobacco growth than silencing the *NtARF17* or *NtARF19* gene and more effectively eradicated the growth enhancement effect of *NtTTG2* overexpression. In contrast, plant growth was not affected by silencing additional nine *NtTTG2*-regulated *NtARF* genes. In double and triple gene silencing combinations, silencing the *NtARF8* gene was more effective than silencing the *NtARF17* or *NtARF19* gene to repress growth as well as nullify growth enhancement. Therefore, the *NtARF8* predominantly cooperated with the *NtARF17* and *NtAFR19* of the *NtTTG2* functional pathway. *NtARF8* also contributed to NtTTG2-regulated seed production as concurrent *NtTTG2* and *NtARF8* overexpression played a synergistic role in seed production quantity, whereas concurrent silencing of both genes caused more severe seed abortion than single gene silencing. In plant cells, the NtTTG2 protein facilitated the nuclear import of NtARF8 as well as increased its function as a transcription activator.

**Conclusions:**

NtARF8 is an integral component of the NtTTG2 functional pathway, which regulates tobacco growth and development.

**Electronic supplementary material:**

The online version of this article (doi:10.1186/s12870-016-0815-3) contains supplementary material, which is available to authorized users.

## Background

TRANSPARENT TESTA GLABRA (TTG) proteins are essential regulators of plant trichome and seed development [1, 2] and have been extensively studied in terms of the functional regulation [3–5]. TTGs are characterized by the presence of the WD40 motif, which comprises the conserved tryptophan (W) and aspartic (D) dipeptide within a length of approximately 40 amino acid residues [6–11]. With WD40, TTGs can directly interact with various types of proteins or serve as interchangeable substrate adaptors that influence protein-protein interactions [11, 12] and therefore are capable of regulating both plant development and immunity [4, 10, 13, 14]. Depending on the biochemical characteristics of cooperative partners, TTGs possess different functions relating to development and immune response [4, 8, 10, 15].

Tobacco (*Nicotiana tabacum* L.) NtTTG1 and NtTTG2 share a high similarity and four WD40 repeats [4, 10]. The WD40 domain enables NtTTG1 to interact with elicitin protein ParA1, which is produced by an oomycete pathogen and induces hypersensitive cell death in plants [10]. The NtTTG1-ParA1 interaction is essential for the induction of programmed cell death initially in leaf trichomes and then in mesophylls, which in turn results in plant resistance to different pathogens [10]. In contrast, NtTTG2 suppresses pathogen resistance in tobacco by indirectly modulating the subcellular localization of protein, NONINDUCER OF PATHOGENESIS-RELATED GENES1 (NPR1) [4], which is a transcription activator of immune response genes [16, 17]. NtTTG2 does not interact with NPR1, but sequesters NPR1 from the nucleus, thereby preventing NPR1 from transcriptionally regulating immune responses [4]. In another study paradigm, AtTTG1 of *Arabidopsis thaliana* Johannes Thal interacts with the bHLH transcription factor GL3, whereas its heterogenous binary complex further interacts with the MYB transcription factor GL1 to form a WD40-bHLH-MYB triplet, which regulates trichome development [12, 18]. These findings suggest that TTGs regulate plant development or immunity by either directly or indirectly interacting with their functional partners.

We have elucidated the developmental function of NtTTG2 by analyses of *NtTTG2*-overexpressing (*TTG2*^+^) and *NtTTG2* silencing (*TTG2*i) transgenic tobacco lines compared to wild-type (WT) plants or a transgenic control line [4]. The transgenic control line WT:*RFP*, which harbors the gene encoding red-fluorescent protein (RFP), resembles the WT in terms of vegetative growth, seed production, and related physiological responses such as floral anthocyanin synthesis and flower colorization [19]. In contrast, growth and development are greatly enhanced in *NtTTG2*-overexpressing *TTG2*^*+*^:*RFP* lines [19], which accumulate the NtTTG2-RFP fusion protein in both the cytoplasm and nucleus [4]. The *NtTTG2*-confered traits of WT plants can be impaired by hairpin-based posttranscriptional gene silencing [4].

We further demonstrate that the NtTTG2 functional pathway for developmental regulation associates with the components of the auxin signaling pathway [19]. In particular, auxin-responsive genes have been identified by *de novo* assembly of the transcriptome of the WT, *TTG2*^+^, and *TTG2*i plants with a common tobacco variety NC89 background following RNA-Seq analyses [19]. The tobacco transcriptome contains 303 unigenes that are related to auxin responses, including 40 unigenes that are predicted to encode AUXIN RESPONSE FACTOR (ARF) proteins [19]. The expression levels of 27 putative *NtARF* genes were unrelated to NtTTG2, whereas the other 13 *NtARF* candidates were regulated by NtTTG2 at the transcriptional level [19]. These findings suggest that the function of NtTTG2 in plant development might be related to the auxin signaling pathway.

The auxin signaling pathway regulates various aspects of plant development [20, 21] through the function of ARFs in transcriptional regulation of auxin responses [22–26]. The regulatory consequence to developmental processes is either positive or negative as ARFs may activate or repress auxin-responsive genes [27–29]. In *Arabidopsis*, for example, 23 ARFs are categorized either as transcription activators or repressors [30]. ARF transcription activators are characterized by the presence of several glutamine (Q) residues in the middle region of protein sequence, whereas transcription repressors are serine-rich in the same region [27, 31]. These ARFs target auxin-responsive genes of the *Small Auxin Up RNA* (*SAUR*), Auxin/Indole-Acetic Acid inducible (*AUX/IAA*), and *Gretchen Hagen 3* (*GH3*) gene families [31–33]. Different ARFs regulate the expression of target genes by binding the auxin response elements, TGTCTC, GAGACA [29, 34, 35], or TGTCT [26] that are present in target promoters. These elements have numerous potential combinations with ARFs [4, 19, 30], but only a few auxin-responsive genes have been shown to be targeted and directly regulated by a specific NtARF [26, 31]. Moreover, ARFs are functionally coregulated by various combinations of AUX/IAA proteins [30, 36]. In essence, despite the large number of ARFs characterized in *Arabidopsis* and other species, direct targets of ARFs and the individual and combinatorial roles remain largely unknown, particularly in species other than *Arabidopsis*.

The purpose of the present study was to identify *NtARF* genes that are associated with the developmental role of NtTTG2, and to elucidate the functional relationship between NtTTG2 and relevant *NtARF*s in tobacco. We focused on 13 *NtARF* candidates that have been previously demonstrated to be regulated by NtTTG2 for expression in tobacco [19]. We followed the accepted nomenclature in designating *NtARF* gene candidates as number-suffixed *ARF*s based on the highest similarities with the corresponding orthologs of different plant species.

Previously, a posttranscriptional gene silencing (VIGS) system was developed with DNA components of the monopartite *Begomovirus* sp. *tobacco curly shoot virus* [37]. We have used the TCSV VIGS system to silence *NtTTG1* [10] and immunity-regulatory genes [38] in tobacco. In the present study, we employed the same system to silence the *NtARF* genes under backgrounds of WT:*RFP*, *TTG2*^+^:*RFP*, and *TTG2*i. Based on the gene silencing effects, we propose that NtTTG2 predominantly regulates NtARF8, followed by NtARF17 and NtARF19, to control vegetative growth and seed production in tobacco.

## Results

### *NtTTG2* responds to auxin but does not affect endogenous auxin levels

**Fig. 1:**
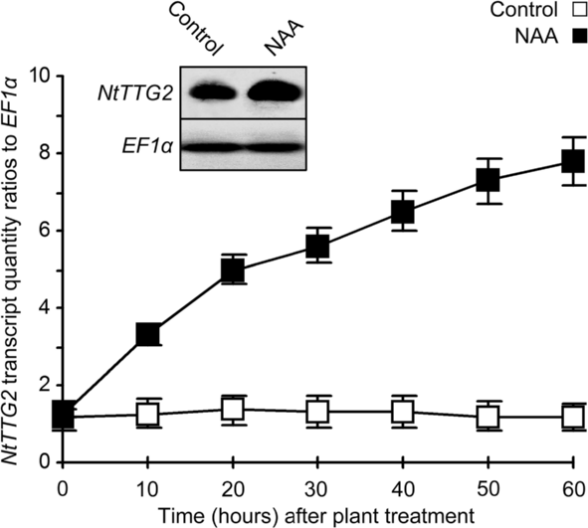
The expression of *NtTTG2* in response to synthetic auxin NAA. Plants were treated by spraying over tops with a surfactant solution in control or with an NAA solution containing the surfactant. Relative levels of *NtTTG2* expression were quantified by RT-qPCR as the transcript quantity ratios to the constitutively expressed *EF1α* gene used as a reference. Data shown are mean values ± standard errors from mean (SEMs) of results obtained from three experimental replicates, i.e., the number of replicates (*n*) = 3. Inset is Northern blot hybridization performed at 60 h after plant treatment

To infer the functional relationship between NtTTG2 and auxin, we analyzed *NtTTG2* expression in leaves of WT tobacco treated with an aqueous solution containing a surfactant and a synthetic auxin, 1-naphthaleneacetic acid (NAA), and with the surfactant solution as control. The NAA treatment highly induced *NtTTG2* expression based on quantitative real-time RT-PCR (RT-qPCR) analysis performed at a 10-min interval in 60 h after plant treatment (Fig. 1, curves). In the period, quantities of *NtTTG2* transcript were increased by 4–6-fold following the NAA treatment compared to the steady-state transcript level of the control (Fig. 1, curves). Marked enhancement of *NtTTG2* expression was confirmed by Northern blotting that was performed at 60 h after plant treatment. In Northern blotting, the *NtTTG2* transcript was detected at a steady-state level compared to the constitutively expressed *EF1α* gene in control plants, whereas *NtTTG2* was upregulated after NAA treatment (Fig. 1, inset).

We measured the endogenous concentrations of IAA in leaves of WT:*RFP*, *TTG2*^*+*^:*RFP*, and *TTG2*i plants. The *TTG2*^*+*^:*RFP* line showed the best developmental traits with the highest level of *TTG2* overexpression, and the *TTG2*i line displayed the highest degrees of *NtTTG2* silencing and developmental compromise [4, 19]. No distinct differences in endogenous IAA content in the aerial organs were observed among the three tobacco genotypes were observed. In all plants, IAA concentrations were detected at basal levels of approximately 70, 150, and 230 ng/g fresh weight of leaves, flowers, and immature fruits (capsules), respectively (Additional file 1: Figure S1). Therefore, *NtTTG2* is responsive to exogenous auxin application, whereas no effect was observed using endogenous auxin content. Because NtARFs are essential for auxin signaling and function of downstream processes of auxin biosynthesis [20, 31], we considered NtTTG2-regulated *NtARF* candidates [19] with respect to the functional connection between NtTTG2 and auxin signaling.

### The 13 NtTTG2-regulated *NtARF* candidates represent 12 *NtARF* genes

According to conventional nomenclature of unigenes identified by the RNA-Seq technique [39], NtTTG2-regulated *NtARF* candidates present in the tobacco transcriptome were specified with unigene codes [19]. For the sake of convenient descriptions, we designated *NtARF* unigenes as conventional gene symbols, with *NtARF* suffixed by numbers. Based on the highest similarities of unigenes with *NtARF* orthologs previously identified in different plant species, the 13 NtTTG2-regelated *NtARF* unigenes represented 12 *NtARF* genes because two unigenes were classified under the same *NtARF* gene (Additional file 2: Table S1). Sequence similarities were considered with tobacco species as first priority over other plants such as Arabidopsis and tomato (*Solanum lycopersicum* L.). The 12 NtTTG2-regulated tobacco *NtARF* genes were thus designated as *NtARF1*, *NtARF2*, *NtARF5*, *NtARF6L* (*NtARF6-Like*), *NtARF8*, *NtARF9*, *NtARF11*, *NtARF16* to *NtARF19*, and *NtARF19L* (*NtARF19-like*) (Additional file 2: Table S1). Similarities in nucleotide sequences were easily compared with published *ARF* homologs by using BLAST.

### The 12 *NtARF*s fall into NtTTG2-upregulated and -downregulated groups

**Fig. 2:**
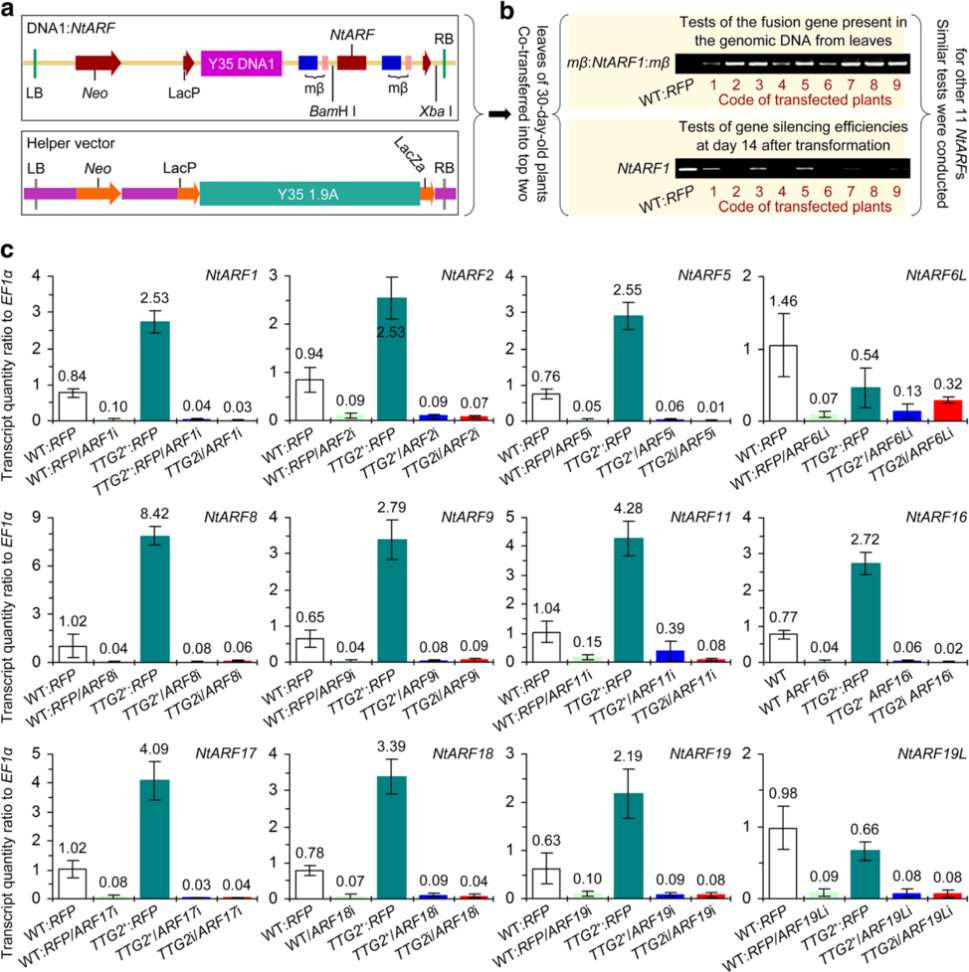
VIGS manipulations of *NtARF* genes and gene silencing efficiencies. **a** Diagrams of the two-component gene-silencing constructs. **b** Technical scheme. The two gel panels represent one of six experimental repeats each containing nine transfected WT:*RFP* plants tested in comparison with WT:*RFP*. Top gel panel is the result of PCR analysis of genomic DNA, and lower gel panel is the result of RT-PCR analysis. Codes of transfected plants are arbitrary. **c** Relative levels of different *NtARF*s in leaves of plants indicated under the corresponding bar graphs. The expression level of each *NtARF* was quantified by RT-qPCR as the transcript quantity ratio of the *NtARF* gene to *EF1α* used as a reference gene based on its constitutive expression. Data shown are mean values ± SEM bars (*n* = 6 experimental replicates)

To elucidate the functional relationship between NtTTG2 and *NtARF* genes, the 12 NtTTG2-regulated *NtARF*s were silenced under backgrounds of WT:*RFP*, *TTG2*^*+*^, and *TTG2*i, followed by assessments of gene expression levels in leaves of distinct plant genotypes. The VIGS system previously developed with DNA components of TCSV [37] and recently used by our group to silence *NtARF8* [38] was employed in the present study to construct silencing units of an additional 11 *NtARF* genes (Fig. 2a). In parallel experiments, *NtARF* gene silencing constructs was used to transfect tobacco plants, followed by verification of transgene integration into the plant genome (Fig. 2b). Then, relative levels of *NtARF* gene expression were quantified by RT-qPCR, which varied with the genetic background of each plant (Fig. 2c).

The elaborate experimental protocol resulted in high gene silencing efficiencies, as shown by marked decreases in the quantities of the corresponding gene transcripts as detected by semi-quantitative RT-PCR (Fig. 2b) and RT-qPCR (Fig. 2c) analyses. All 12 NtTTG2-regulated *NtARF* genes were silenced at high efficiencies (>95 %) compared to the steady-state expression levels under WT:*RFP* background (Fig. 2c). Under the *TTG2*^+^:*RFP* background, *NtARF6L* and *NtARF19L* had fewer transcripts, but the additional ten genes (*NtARF1*, *NtARF2*, *NtARF5*, *NtARF8*, *NtARF9*, *NtARF11*, *NtARF16*, *NtARF17*, *NtARF18* and *NtARF19*) accumulated more transcripts than the WT:*RFP* plant. These genes showed opposite behaviors under a *TTG2*i background compared to *TTG2*^+^:*RFP* (Fig. 2c). Thus, *NtARF1*, *NtARF2*, *NtARF5*, *NtARF8*, *NtARF9*, *NtARF11*, *NtARF16*, *NtARF17*, *NtARF18* and *NtARF19* comprised a group of NtTTG2-upregulated *NtARF* genes, whereas *NtARF6L* and *NtARF19L* represented the downregulated group. In the ten NtTTG2-upregulated *NtARF*s, *NtARF8* displayed the highest degree of expression enhancement by NtTTG2 (Fig. 2c). On the basis of downregulation by *TTG2*i, expression levels of NtTTG2-upregulated *NtARF*s were further reduced in double gene silencing *TTG2*i/*ARF*i lines. Inversely, *NtARF* expression levels were partially recovered in *TTG2*^+^:*RFP/ARF*i plants, which resulted from the silencing *NtARF*s under a TTG2^+^:*RFP* background (Fig. 2c). The high levels of single *NtARF* gene silencing and the synergistic effects of *NtTTG2* and *NtARF* double modifications of *NtARF* expression may be further investigated in future studies to elucidate the roles of *NtARF*s in NtTTG2-regulated plant growth and development.

### *NtARF8*, *NtARF17*, and *NtARF19* contribute to *NtTTG2*-regulated plant growth

**Fig. 3:**
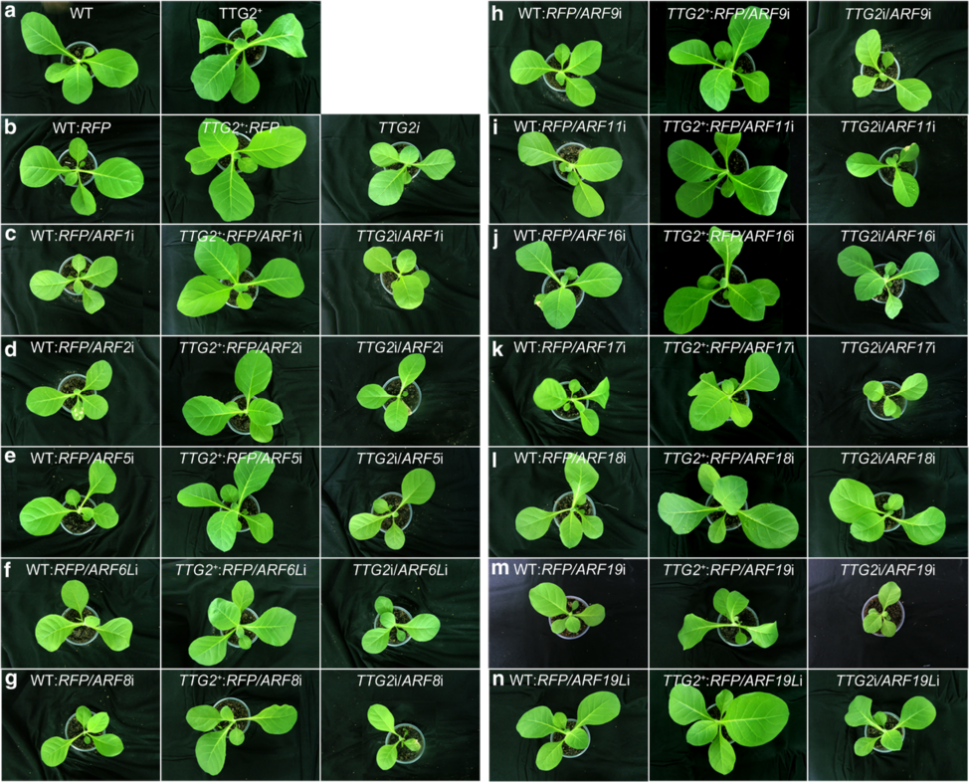
Plants photographed two weeks after transfection for gene silencing manipulations. WT (or WT:*RFP*), *TTG2* ^*+*^ (or *TTG2* ^+^:*RFP*), and *TTG2*i plants (**a**, **b**) were used as backgrounds for comparisons of the phenotypic effects of single *NtARF* gene silencing and concomitant silencing of *TTG2* and an *NtARF* gene (**b**–**n**)

**Fig. 4:**
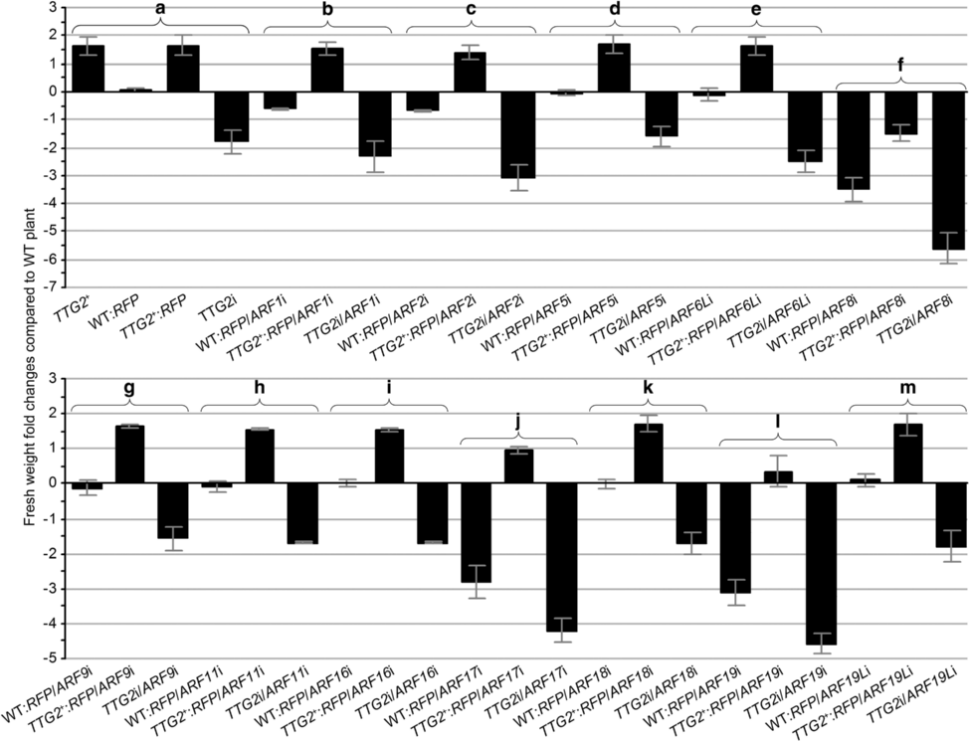
Fold changes in fresh weight of differently modified plants in contrast to the WT plant. Fold changes in fresh weight of differently modified plants in contrast to the WT plant. WT:*RFP*, *TTG2* ^*+*^, *TTG2* ^+^:*RFP*, and *TTG2i* (**a**) were used as backgrounds for comparisons of the phenotypic effects of single *ARF* gene silencing and concomitant silencing of *TTG2* and an *ARF* gene (**a**–**m**). Data shown are mean values ± SEM bars (*n* = 6 experimental replicates)

Recently we characterized the role of NtTTG2 in tobacco growth based on increases of the biomass (fresh weight) of plants grown in a medium and in pots, respectively [19]. The contributions of the different *NtARF* genes to NtTTG2-regulated tobacco growth were evaluated by direct observation of growth rates (Fig. 3) and by comparing fresh weight changes (Fig. 4) of the different plants at 15 days after transfection. No apparent differences in growth rates between WT:*RFP* and WT were observed, neither between *TTG2*^+^ and *TTG2*^+^:*RFP* plants (Fig. 3a compared to Fig. 3b), thereby confirming the absence of any phenotypic effects in the controls. The potential effects of *NtARF*s on NtTTG2-regulated plant growth were assessed by imaging plants with and without *NtARF* gene silencing manipulations (Fig. 3 c–n compared to Fig. 3b). The low degree of plant growth repression was caused by silencing *NtARF1*, *NtARF2*, *NtARF5*, *NtARF6L*, and *NtARF9*, respectively (Fig. 3c–f, h compared to Fig. 3b). A marked repression of growth was observed with *NtARF8*, *NtARF17*, or *NtARF19* silencing. Independent silencing of the three *NtARF*s caused a marked reduction in plant size under the WT:*RFP* background, further reduction in *TTG2*i plant size, and a decrease in plant growth enhancement by *TTG2*^+^ (Fig. 3g, k, and m compared to Fig. 3b). In contrast, silencing *NtARF11*, *NtARF16*, *NtARF18*, or *NtARF19L* did not cause evident changes in plant growth rates under the same genetic background (WT:*RFP*, *TTG2*^*+*^:*RFP*, or *TTG2*i; Fig. 3i, j, l, and n compared to Fig. 3b).

The observed differences in plant growth extents were confirmed by quantifying fold-changes in fresh weight of transfected plants compared to control plants (Fig. 4). Similar weight changes (~1.7-fold) were observed in *TTG2*^*+*^ and *TTG2*^*+*^:*RFP* plants, whereas the weight of WT:*RFP* plants showed minimal change compared to that of the WT (Fig. 4a). These plants were used as basis in assessing the effects of *NtARF* silencing under the corresponding genetic backgrounds (Fig. 4b–m). A less than 0.5-fold decrease or no evident changes were observed with silencing of *NtARF1*, *NtARF2*, *NtARF5*, *NtARF6L*, *NtARF9*, *NtARF11*, *NtARF16*, *NtARF18* or *NtARF19L* under the WT:*RFP* background (Fig. 4b–e, g–k, and m compared to Fig. 4a). Similar results were obtained with silencing any of these *NtARF* genes under the backgrounds of *TTG2*^*+*^ (or *TTG2*^*+*^:*RFP*) and *TTG2*i (Fig. 4b–e, g–k, and m compared to Fig. 4a). In contrast, plant weight showed a 3.6-fold, 2.8-fold, and 3.1-fold decrease, respectively, when *NtARF8*, *NtARF17*, and *NtARF19* were silenced under the WT:*RFP* background (Fig. 4f, j, l). Moreover, a 2-fold decrease in plant weight was observed after the independent silencing of *NtARF8*, *NtARF17*, and *NtARF19* under the *TTG2*i background (Fig. 4, f, j, l) compared to that observed under WT:*RFP* (Fig. 4a). When *NtARF17* and *NtARF19* were silenced under a *TTG2*^*+*^ background, the extent of TTG2^+^-conferred plant growth enhancement was reduced by 0.53-fold (53 %) and 1.5-fold, respectively (Fig. 4, j and l compared to Fig. 4a). A stronger impairment to *TTG2*^*+*^ plant growth was observed with *NtARF8* silencing, which totally cancelled the growth enhancement effect and further decreased fresh weight by 1.3-fold (Fig. 4f compared to Fig. 4a). These findings suggest that *NtARF8*, *NtARF17*, and *NtARF19* are major coregulators of the NtTTG2 functional pathway for the plant growth, whereas *NtARF8* plays a predominant role.

### *NtARF8* is the predominant interactor of *NtARF17* and *NtARF19* in NtTTG2-regulated plant growth

**Fig. 5:**
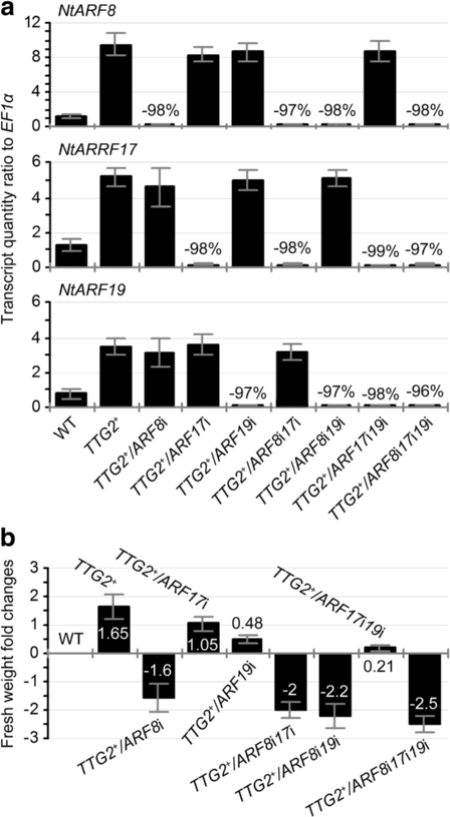
Concurrent *NtARF8*, *NtARF17*, and *NtARF19* silencing manipulations and the subsequent effects on plant growth. **a** *NtARF8*, *NtARF17*, and *NtARF19* silencing efficiencies. Gene silencing was performed under *TTG2* ^+^ background to evaluate the combinational effects of *NtARF8*, *NtARF17*, and *NtNAR19* on the role of *NtTTG2* in the plant growth. Gene expression levels were determined by RT-qPCR as transcript quantity ratios to *EF1α*. Percent reductions of *NtARF* transcript quantities in the different gene silencing manipulations were quantified in relative to *TTG2* ^+^ background. Gene expression levels under backgrounds of WT, *TTG2* ^+^ only, and *TTG2* ^+^ with a single *NtARF* gene silencing are shown for comparisons of plant growth changes. **b** Plant fresh weight alterations by *NtTTG2* overexpression and *NtARF* silencing compared to WT background. Data shown in **a**, **b** are mean values ± SEM bars (*n* = 6 experimental replicates)

To confirm the roles of *NtARF8*, *NtARF17*, and *NtARF19* in the NtTTG2 functional pathway, we investigated changes in fresh weight of *TTG2*^*+*^ plants following transfection of different leaves with each of the three *NtARF* VIGS constructs and with double and triple combinations at various time points. In all transfected plants, the three genes were silenced by >95 % and in an independent manner (Fig. 5a). For example, silencing *NtARF8* did not affect the expression levels of *NtARF17* and *NtARF19*, whereas concurrent silencing of *NtARF8* and *NtARF17* did not affect *NtARF19* expression. In addition, the expression levels of *NtARF8*, *NtARF17*, and *NtARF19* were simultaneously downregulated only with the triple gene silencing manipulation in single plants (Fig. 5a).

Plant weight was determined at 14 days after the last round of transfection. Based on fold-changes in fresh weight in *NtARF*-silenced *TTG2*^+^ lines compared to the WT plant, double or triple gene silencing manipulations acted synergistically in impairing *TTG2*^*+*^-conferred plant growth enhancement after single gene silencing (Fig. 5b). The most extensive compromise in plant growth enhancement was observed after silencing *NtARF8* under double or triple gene silencing conditions. In double combinations, *NtARF8* silencing further reduced plant weight by 1.9- and 4.9-fold based on independent reductions by *NtARF17* and *AFR19* silencing. However, silencing of *NtARF17* and *NtARF19* concurrently with *NtARF8* caused an additional 25 % and 38 % decrease in plant weight compared to silencing *NtARF8* alone. The triple gene silencing manipulation further reduced plant weight by 0.56–, 3.5-, and 6.1-fold (56 %, 350 %, and 610 %) over the single gene silencing effects of *NtARF8*, *NtARF17*, and *NtARF19*, respectively. Clearly, silencing of *NtARF8* was more repressive to plant growth than silencing *NtARF17* or *NtARF19*. Furthermore, *NtARF8* was expressed in the growing leaves of 10–50-day-old plants, much higher than that observed in *NtARF17* and *NtARF19* (Additional file 3: Figure S2). During plant growth, the ratios of *NtARF8*, *NtARF17*, and *NtARF19* transcripts to the reference gene (*EF1α*) were 1.02–1.30, 0.76–1.00, and 0.70–0.93, respectively. These findings suggest that *NtARF8* plays a predominant role in the genetic cooperation with *NtARF17* and *NtARF19* in order for NtTTG2 to regulate plant growth.

### *NtARF8* contributes to NtTTG2-regulated seed production

**Fig. 6:**
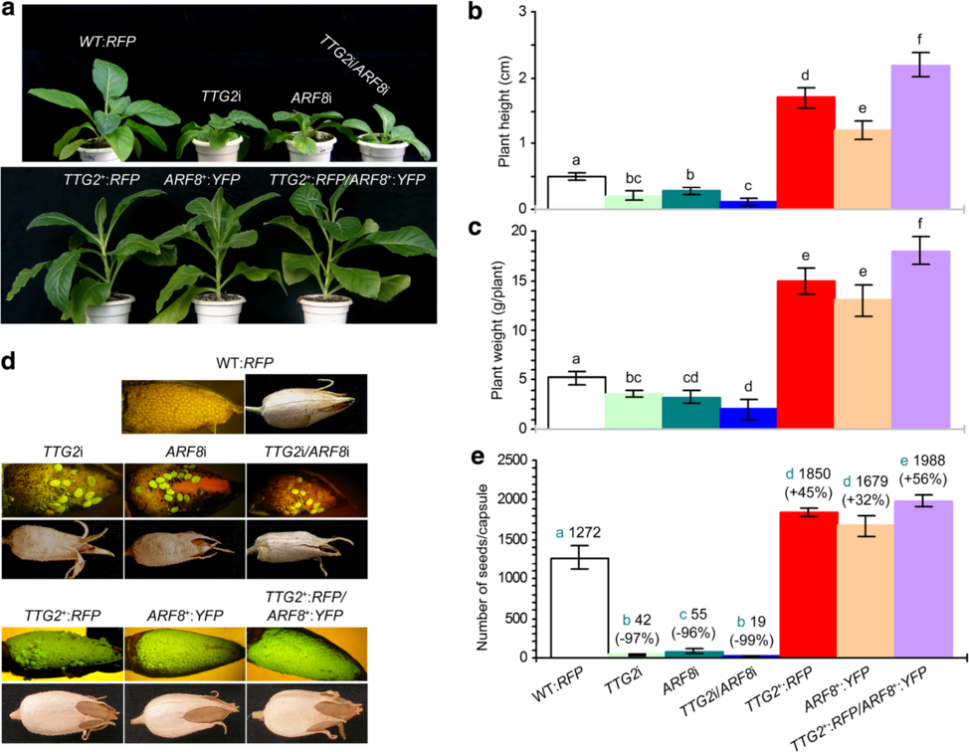
The effects of concurrent *TTG2* and *ARF8* modifications on plant growth and seed production. Plants were grown in the greenhouse (plants used in other experiments were grown in the chamber). **a** Appearance of 50-day-old plants. **b** Plant height scoring. **c**, **d** Seed production and fruit appearance. Data shown in **b**, **c**, **e** are means ± SEM bars; different letters indicate significant differences among plant genotypes (*n* = 6; *P* < 0.01)

Similar to foliar expression (Fig. 5a), *NtARF8* is also expressed in other organs, including immature fruits, in an NtTTG2-dependent manner (Additional file 4: Figure S3). Thus, *NtARF8* may participate in the NtTTG2-regulated developmental process in addition to the vegetative growth. We tested this hypothesis by analyzing the functional relationship between the *NtTTG2* and *NtARF8* genes for tobacco seed production, which is an important developmental trait that is regulated by *Nt*TTG2 [19]. We confirmed the synergistic effect of concurrent *NtTTG2* and *NtARF8* silencing on the vegetative growth of elder plants (Fig. 6a–c) compared to that of younger plants (Figs. 3g and 4f). We also confirmed the genetic cooperation of *NtTTG2* and *NtARF8* by evaluating the effect of concurrent gene overexpression on plant growth (Fig. 6a–c). The transformation of WT and *TTG2*^*+*^:*RFP* plants with *NtARF8*, which was fused to the gene encoding yellow-fluorescent protein (YFP), resulted in the transgenic lines, *ARF8*^+^:*YFP* and *TTG2*^*+*^:*RFP*/*ARF8*^*+*^:*YFP*, respectively. Five *ARF8*^+^:*YFP* lines and five *TTG2*^*+*^:*RFP*/*ARF8*^*+*^:*YFP* lines were characterized based on plant growth enhancements, as indicated by the independent and concurrent overexpression of *NtARF8* and *NtTTG2* (Additional file 5: Figure S4).

We compared an *ARF8*^+^:*YFP* line and a *TTG2*^*+*^:*RFP*/*ARF8*^*+*^:*YFP* line with WT:*RFP*, *TTG2*i, *NtARF8*i, *TTG2*i/*NtARF8*i, and *TTG2*^*+*^:*RFP* plants in terms of seed production quantity. Although seed densities in fruits were similarly observed in some genotypes (Fig. 6d), the number of seeds in a single fruit significantly varied with genetic modification of *NtTTG2* and *NtARF8* (Fig. 6e). Independent silencing of *NtTTG2* and *NtARF8* resulted in acute seed abortion, whereas more than 97 % and 96 % seeds were aborted (Fig. 6e), respectively, in *NtTTG2*i and *NtARF8*i plants compared to that observed in WT:*RFP*. More severe seed abortion resulted from concurrent *NtTTG2* and *NtARF8* silencing, which further reduced seed number per fruit over the single gene silencing effects (Fig. 6e). Seed abortion lead to shrunken fruits, which is as an evident phenotype that was associated with *NtTTG2* and *NtARF8* silencing compared to the observed normal seed appearance with WT:*RFP* (Fig. 6d). In contrast, the independent overexpression of *NtTTG2* or *NtARF8* substantially promoted seed production, causing a 45 % and 32 % increase in seed numbers in single fruits (Fig. 6e), along with the formation of plump fruits (Fig. 6d). An enhancement of seed production was observed with concurrent *NtTTG2* and *NtARF8* overexpression, which resulted in an 11 % and 24 % increase in seed number compared to the effects of the independent overexpression of *NtTTG2* and *NtARF8*, respectively (Fig. 6e). These findings suggest that *NtARF8* is an essential component of NtTTG2-regulated seed production in tobacco. This notion is in agreement with the altered NtTTG2 expression in young fruits (Additional file 4: Figure S3) and additionally confirms the role of NtTTG2 in seed production [19].

### NtARF8 is a functional transcription activator of illustrational target gene

**Fig. 7:**
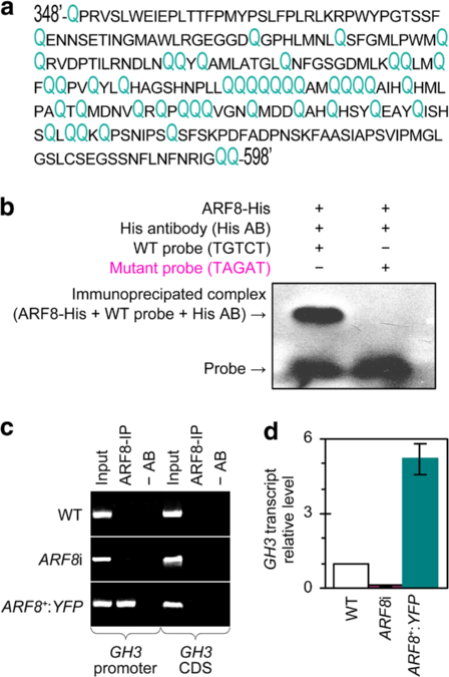
Partial sequence characterization and assays of the NtARF8 protein as a transcription activator. **a** Partial sequence of the predicted NtARF8 protein. The NtARF8 sequence contains 843 amino acids and only the middle region is presented to indicate glutamine (Q) abundance as the characteristics of ARF transcription activators. **b** An EMSA assay of the NtARF8-His (ARF8-His) fusion protein, the WT probe for the auxin-responsive TGTCT element, which is present in the *GH3* gene promoter, and the mutant probe. **c** ChIP PCR conducted with chromatins isolated from leaves of plants indicated on left, the specific YFP antibody, and primers specific to the promoter and CDS of *GH3*. Bands in gel indicate products of PCR conducted with DNA templates from the precipitated complex consisting of chromatin fragments and the NtARF8-YFP fusion protein (ARF8-IP), the mock precipitation in the absence of the antibody (−AB), and chromatins before immunoprecipitation (input), respectively. **d** *GH3* expression levels quantified by RT-qPCR. The WT transcript quantity ratio of *GH3* to *EF1α* was defined as 1 and scored accordingly for *ARF8*i and *ARF8* ^*+*^:*YFP*. Data shown are mean values ± SEM bars (*n* = 3 experimental replicates)

The NtARF8 protein (GenBank PMID 24875793) is the predicted protein of the *NtARF8* gene, which we recently cloned from the common tobacco variety, NC89 [4, 38]. The putative NtARF8 protein is highly identical at the amino acid level to well-characterized homologs (Additional file 6: Figure S5) and shares the conserved Q tract at the middle region (Fig. 7a), which characterizes ARF transcription activators [27, 31]. The Q-rich region spans residue sites 348 to 598 in the NtARF8 sequence (Fig. 7a), which consists of 843 amino acids in total. Based on these structural characteristics, NtARF8 is a potential transcription activator.

To validate this prediction, we used the *GH3* (*Nt-gh3*) gene to illustrate the role of NtARF8 in transcriptional activation. The rationale behind this experiment is that *GH3* is an auxin-responsive gene in tobacco [22, 40] and was recently detected in the NtTTG2-regulated profile of the tobacco transcriptome [19]. *GH3* expression is *NtTTG2*-dependent, enhanced by *NtTTG2* overexpression, but repressed by *NtTTG2* silencing, whereas *NtARF8* performs as an *NtTTG2* gene, affecting *GH3* expression (Additional file 7: Figure S6). Moreover, typical auxin response element TGTCT [26] exists as two repeats (409–413 and 798–802) in the *GH3* promoter (GenBank Accession number AJ620494). In an electrophoresis mobility shift assay (EMSA), this element prepared as a WT probe was shown to bind to the NtARF8-His fusion protein, but not the mutant ACAGC (Fig. 7b).

Chromatin immunoprecipitation (ChIP) using chromatin isolated from WT:*RFP*, *NtARF8*i, and *NtARF8*^*+*^:*YFP* leaves was performed to test whether NtARF8 could bind to the *GH3* promoter. The specific RFP antibody was used to identify the target DNA composition of the chromatin sample, and the target DNA was assumed to combine with the ARF8-RFP fusion protein present in the same chromatin sample. The precipitated DNA was quantified by PCR in parallel with two pertinent controls. One was the input reference, in which a part of the chromatin sample was not subjected to immunoprecipitation. Another was the antibody-absent reference (−AB), in which a different part of the chromatin sample was subjected to the standard immunoprecipitation procedure in the absence of the antibody. These DNA samples were PCR amplified using primers that were specific to the promoter or coding sequence (CDS) of the *GH3* gene. Figure 7c shows that no *GH3* CDS DNA was detected in the chromatin of the three plants, whereas the *GH3* promoter was detected only in the *NtARF8*^+^:*YFP* chromatin samples, thereby suggesting the specific binding of NtARF8 to the promoter. Similarly, the *GH3* gene was expressed in *ARF8*^+^:*YFP* or WT:*RFP*, but not in *ARF8*i plants, and the gene transcript was 5-fold higher in *ARF8*^+^:*YFP* plants than in WT:*RFP* plants (Fig. 7d). These findings indicated that NtARF8 indeed is a transcription activator of the illustrational target gene.

### NtTTG2 facilitates nuclear localization of NtARF8

**Fig. 8:**
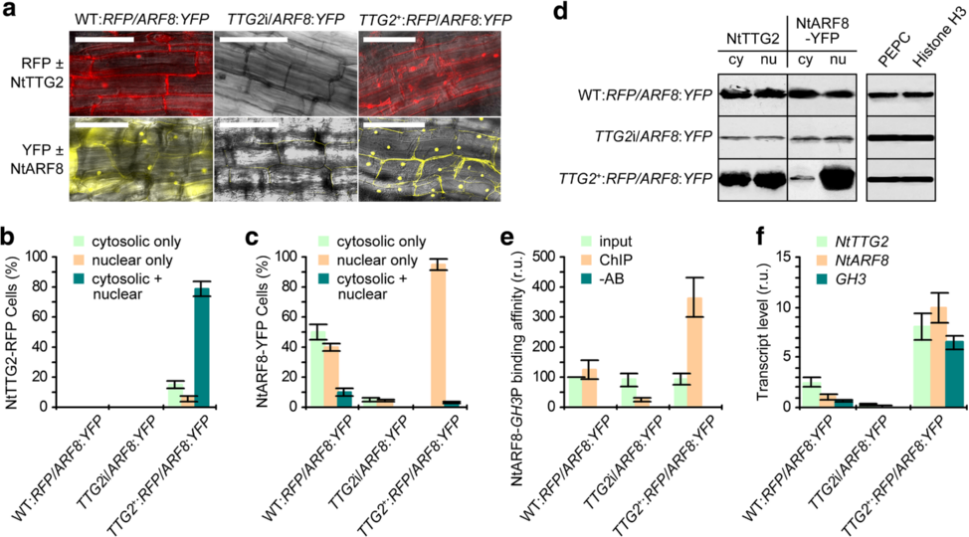
The effects of altered NtTTG2 protein quantities on subcellular localization of the NtARF8 protein and associated gene expression. **a** Confocal microscopic imaging of roots transformed with the *ARF8-YFP* fusion gene. Scale bars = 100 μm. **b**, **c** Percentage of root cells showing TTG2-RFP and ARF8-YFP fluorescence signals, respectively, only in the cytoplasm, only in the nucleus, or in both compartments. Cells in totally 10 fields of vision were counted for RFP and YFP fluorescence signals, respectively. Data shown are mean values ± SEM bars (*n* = 360–390 cells). **d** Western blots showing protein levels in cytoplasmic (cy) and nuclear (nu) fractions isolated from leaf cells. PEPC and histone H3 were used as cytosolic and nuclear markers, respectively. **e** ChIP PCR analyses to quantify levels of the affinity for NtARF8-YFP binding with the *GH3* promoter. **f** Expression levels of related genes quantified by RT-qPCR as the transcript quantity ratios to *EF1α*. Data shown in **e**, **f** are mean values ± SEM bars (*n* = 3 experimental replicates)

To infer how NtTTG2 regulates the function of NtARF8 as a transcription activator, we tested whether both proteins directly interacted. No interaction was detected by using a yeast two-hybrid assay or by bimolecular fluorescence complementation (Additional file 8: Figure S7). Then, we monitored the subcellular distributions of both NtTTG2-RFP and NtARF8-YFP fusion proteins after transfecting the roots of WT:*RFP*, *TTG2*i, and *TTG2*^+^:*RFP* plants with the *NtARF8*:*YFP* fusion gene. Fluorescence imaging of transfected root cells indicated that the subcellular localization of NtARF8 changed with NtTTG2 quantity alterations (Fig. 8a). Fluorescence was absent under the *TTG2*i background, whereas high levels of RFP fluorescence was observed in the cytoplasm and nucleus of the WT:*RFP* cells. Under the *TTG2*^+^:*RFP* background, strong fluorescence of the NtTTG2-RFP fusion protein was observed in both the cytoplasm and nucleus (Fig. 8a). The three genetic backgrounds had different impacts on the subcellular localization of the NtARF8-YFP fusion protein. This protein was observed with *TTG2*i, at a high level of accumulation in the WT:*RFP* and *TTG2*^+^:*RFP* roots (Fig. 8a). Similar to the dual localization of NtTTG2, NtARF8-YFP was also detected in both the cytoplasm and nucleus of WT:*RFP* cells. In *TTG2*^+^:*RFP*, NtARF8-YFP was predominantly detected in the nucleus (Fig. 8a).

To quantify the effect of altered NtTTG2 levels on the subcellular localization of NtARF8, we counted the number of root cells showing fluorescence of NtTTG2-RFP (Fig. 8b) or NtARF8-YFP (Fig. 8c) only in the cytoplasm, only in the nucleus, and simultaneously in both. Our observations confirmed the dual localization of NtTTG2-RFP in both the cytoplasm and nucleus (Fig. 8c). The number of cells with cytoplasmic, nuclear, or dual location of NtARF8-YFP was determined by quantities of NtTTG2 under the different genetic backgrounds (Fig. 8c). NtARF8-YFP was detected only in the cytoplasm and only in the nuclei at approximately equivalent proportions (40 %–50 %) of WT:*RFP* cells, whereas NtARF8-YFP was exclusively localized to nuclei in more than 95 % of the *TTG2*^+^:*RFP* cells. In both cytoplasmic and nuclear spaces, NtARF8-YFP was observed at small amounts or was not detected under the *TTG2*i background (Fig. 8c). These data suggest that overexpressed NtTTG2 promoted NtAFR8 localization to the nucleus.

### NtTTG2-facilitated nuclear localization of NtARF8 enhances its role as a transcriptional activator

The results of root cell fluorescence imaging (Fig. 8a–c) were in agreement with the findings of immunoblotting analysis of proteins from cytoplasmic and nuclear fractions of leaf cells (Fig. 8d). Cytoplasmic and nuclear proteins were analyzed together with phosphoenolpyruvate carboxylase (PEPC) and histone H3, which were used as cytoplasmic and nuclear markers, respectively [4, 41]. A group of protein blots were hybridized with the NtTTG2 antibody, which was generated by immunizing a New Zealand white rabbit in our previous study [4]. This hybridization detected remarkable quantities of the NtTTG2 protein in WT:*RFP* and *TTG2*^+^:*RFP* plants, although a low protein expression level was observed with *TTG2*i. While similar levels of NtTTG2 were observed in the cytoplasmic and nuclear fractions of WT:*RFP*, a higher level was observed in the nucleus compared to that detected in the cytoplasm of *TTG2*^+^:*RFP* cells (Fig. 8d). An independent group of protein blots were hybridized with a specific commercially manufactured YFP antibody [4]. Hybridization showed that the NtARF8-YFP fusion protein was expressed at similar amounts in the cytoplasm and nucleus of WT:*RFP* cells, whereas it was downregulated in *TTG2*i cells. In *TTG2*^+^:*RFP* cells, a high level of NtARF8-YFP was detected in the nucleus, whereas a lower level was observed in the cytoplasm (Fig. 8d). These findings confirmed that overexpressed NtTTG2 facilitates in the localization of NtARF8 to the nucleus. These results also suggest that sufficient production and nuclear localization of NtTTG2 are required for the nuclear localization of NtARF8.

Nuclear localization is a prerequisite for transcription factors to function in gene expression regulation [42]. Thus, it is logical to hypothesize that NtTTG2-modulated subcellular localization of NtARF8 is mandatory to affect the expression of the *GH3* gene, which was used as an illustrational target of NtARF8 (Fig. 7b–d). A possible intermediate mechanism is that increased nuclear localization offers more opportunities for the transcription factor to bind to the target promoter. This hypothesis was validated by the ChIP PCR assay and by quantification of gene expression levels in leaves. A foliar ChIP DNA sample was quantified by RT-qPCR in parallel with a DNA sample from the input reference and another DNA sample from the AB-absent reference. By using this elaborate assay, the affinity of NtARF8-YFP binding with the *GH3* promoter (*GH3*P) was quantified as relative amounts of PCR product from quantitative leaf samples that were processed in a uniform reaction volume. Figure 8e shows that a very low degree of ARF8-*GH3*P binding affinity was detected with *TTG2*i/*ARF8*:*YFP* compared to the reference level from input. Inversely, ARF8-*GH3*P binding affinity significantly increased with *TTG2*^*+*^:*RFP*/*ARF8*:*YFP* compared to that observed with WT:*RFP*/*ARF8*:*YFP* background (Fig. 8e). These findings indicate that NtTTG2-facilitated nuclear localization of NtARF8 increases its ability to bind with the *GH3* promoter. *GH3* was thus upregulated in *TTG2*^+^:*RFP* leaves, but its expression was highly repressed in *TTG2*i:*RFP* compared to the observed steady-state expression in leaves of WT:*RFP* plants (Fig. 8f). A similar pattern of foliar expression was observed with *NtARF8* and *NtTTG2* (Fig. 8f). Therefore, NtARF8 and NtTTG2 involve a concerted regulatory mechanism at the transcription level in addition to its concomitant role in subcellular localization.

### *NtTTG2* and *NtARF8* coordinately respond to auxin

**Fig. 9:**
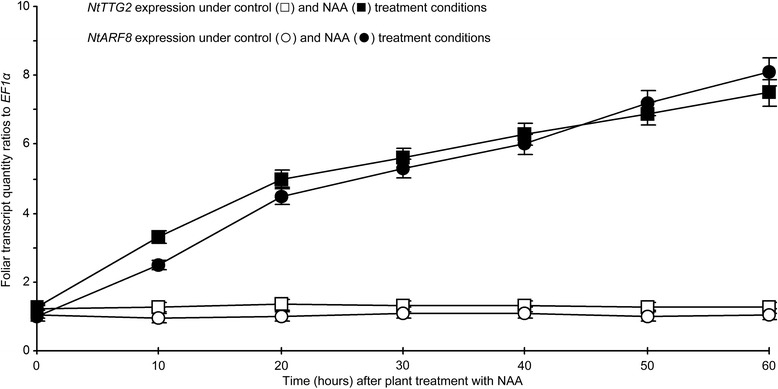
Coordinate responses of *NtTTG2* and *NtARF8* to auxin. WT plants were treated by spraying over tops with a surfactant solution (control) or an NAA solution containing the surfactant. Gene expression in leaves was analyzed at the indicated time points by RT-qPCR using *EF1α* as a reference gene. Data shown are mean values ± SEM bars (*n* = 3 experimental replicates)

The functional coordination of *NtTTG2* and *NtARF8* was further evidenced by the concomitant expression of both genes under two circumstances. First, *NtTTG2* silencing and overexpression resulted in a significant enhancement and repression of *NtARF8* expression, respectively, whereas similar modifications of *NtARF8* did not affect *NtTTG2* expression (Additional file 7: Figure S6). Therefore, *NtTTG2* is indispensable to *NtARF8* expression, implying that *NtTTG2* functions upstream of *NtARF8* in regulating the plant growth and development. Second, *NtARF8* resembled *NtTTG2* in terms of its response to auxin and expression enhancement in 60 h after NAA application to the WT plant (Fig. 9). In a parallel experiment, the amount of *NtARF8* transcripts increased by 6.9 times at 60 h after the NAA treatment compared to the control. At the same time point, *NtTTG2* expression was upregulated 5.3 times compared to the steady-state levels in detected in the control (Fig. 9). The coordinate responses of *NtTTG2* and *NtARF8* to exogenously applied NAA suggest that both genes are concurrently involved in auxin signaling.

## Discussion

Previous studies have shown that TTGs regulate plant developmental processes and/or immune responses by interacting or cooperating with different functional partners [4, 10, 12, 18]. In the *Arabidopsis* trichome developmental model, the functional partners of AtTTG1 include two distinct transcription factors, a bHLH [12] and a MYB [18]. In tobacco, NtTTG1 regulates immune responses by recognizing a biotic elicitor and involving transcription factor, NPR1 [10]. In contrast, NtTTG2 is characteristic of an immunity repressor, which particularly suppresses the NPR1 functional pathway to confer disease susceptibility [4]. NtTTG2 also acts as a positive regulator of development and functions in association with auxin signaling, particularly in relation to the differential expression of 12 *NtARF* genes [19]. In the present study, we elucidated a previously unappreciated mechanism by which NtTTG2 regulates vegetative growth and seed production. This novel mechanism is executed by the genetic cooperation of *NtARF8*, *NtARF17*, and *NtARF19*, which are abundantly expressed in the NtTTG2-upregulated transcriptional profile of auxin-related genes [19] (Additional file 2: Table S1). We further elucidated that NtARF8 plays a predominant cooperative role with NtARF17 and NtARF19 that influences the NtTTG2 functional pathway.

We have shown that ARF8 is a major mechanistic linker between NtTTG2 and auxin signaling in the regulation of tobacco growth and development. Evidence has been provided at the transcriptional and post-transcriptional levels, as well as the cytological scale, particularly with respect to the functional relationship between *NtTTG2* and *NtARF8* genes and proteins. At the transcriptional level, the *NtTTG2* gene is induced by the exogenously applied auxin (Fig. 1), but alterations in the expression levels of *NtTTG2* does not affect the endogenous content of auxin in different plant organs (Additional file 1: Figure S1). Based on the link between *NtTTG2* and auxin signaling, the *NtARF8* gene appears to be the most possible candidate. *NtARF8* is one of newly characterized 12 *NtTTG2*-regulated *NtARF* genes (Additional file 2: Table S1) that were originally identified as 13 putative *ARF* genes in the tobacco transcriptome [19]. The expression of *NtARF8* (comp30272_c0 and comp42904_c0) was upregulated by *NtTTG2* overexpression and downregulated by *NtTTG2* silencing. Under the same conditions, *NtARF17* (comp39443_c0) and *NtARF19* (comp38146_c2) showed a similar behavior as that of *NtARF8*, with lower levels of expression than *NtARF8* but higher than the additional 9 *NtTTG2*-regulated *ARF* genes [19]. These findings suggest that NtTTG2 acts after auxin production to regulate *ARF* gene expression.

Based on the gene silencing effects, 10 and 2 of the 12 characterized *ARF* genes were respectively positively and negatively regulated NtTTG2 (Fig. 2). In the NtTTG2-upregulated genes, *NtARF8* silencing was more effective than silencing *NtARF17* or *NtARF19* to repress the growth and to eradicate the enhanced growth effect of *NtTTG2* overexpression (Figs. 3, 4 and 5). This finding is suggestive of the dominant role of *NtARF8* in relation to *NtARF17* and *NtARF19*. By integrating these findings with those of the gene silencing effects on seed production quantity (Fig. 6), we propose that NtARF8 is an integral component of NtTTG2-regulated plant growth and development. At the molecular level, the functional relationship between NtTTG2 and NtARF8 differs from that of the WD40-bHLH-MYB complex for the trichome development [12, 19], and is also distinct from that of the AtARF8-bHLH interaction for petal growth [8] in *Arabidopsis*. At the phenotypic level, the functional pathway consisting of NtTTG2 and NtARF8 is responsible for vegetative growth and seed production, but whether it also affects other NtTTG2-regulated traits [4, 19] remains to be determined.

At the cytological level, the NtTTG2 protein indirectly regulates the function of NtARF8 as a transcription activator by modulating its localization to the nucleus (Figs. 7 and 8). The function of NtARF8 as a transcription activator has been demonstrated by using *GH3*, which is an NtTTG2-regulated auxin-responsive gene that was previously identified in tobacco [16, 23] (Additional file 7: Figure S6) as an NtARF8 potential target genes. The rationale behind the selection of the tobacco *GH3* gene was based on the stimulatory effect of AtARF8 on the expression of *GH3* orthologs in *Arabidopsis* [35], namely the presence of a typical auxin response element (TGTCT) in the GH3 promoter (GenBank Accession number AJ620494). Based on element recognition by ARF transcription activators [26], the ability of the NtARF8 protein to combine with the *GH3* promoter is in line with *GH3* expression levels that decreased by silencing but increased with *NtARF8* overexpression (Fig. 7). The transcription regulatory activity of NtARF8 requires nuclear import under modulation by the NtTTG2 protein, whereas NtTTG2 fulfill this cytological role depending on its dual location in the cytoplasm and nucleus (Fig. 8). When NtTTG2 remains at the steady-state level under WT background, NtARF8 is also produced at the same steady-state level with the dual location (Fig. 8). This observation implies that the steady-state level of NtARF8 is sufficient in regulating NtTTG2-conferred development. This assumed dynamic relationship between NtTTG2 and NtARF8 is in agreement with the ready-state level of the NtTTG2 protein or the gene transcript [4] (Figs. 1 and 8) and with the basal level of auxin (Additional file 1: Figure S1) under a WT background. With overexpression, however, increased amounts of the NtTTG2 protein result in elevated proportions of NtARF8 localization to the nucleus, accompanied by t *GH3* upregulation (Fig. 8). Thus, the nuclear localization of NtARF8 and its performance to activate target gene expression are essential steps in the NtTTG2 functional pathway. This pathway is executed by a crosstalk with auxin signaling, as evidenced by coordinate responses of *NtTTG2* and *NtARF8* to auxin (Fig. 9) and by the NtTTG2-dependent role of NtARF8 in regulating transcription of the auxin-response gene (Figs. 7 and 8).

The tobacco *GH3* gene was used only as an example of an NtARF8 target gene to elucidate the function of NtARF8 as a transcription activator. We did not intend to elucidate whether *GH3* contributes to NtTTG2-conferred tobacco growth and development. *GH3* genes encode IAA-conjugating enzymes that presumably terminate the auxin signal for plants to retrieve auxin hypersensitivity or elicit biotic stress responses during plant growth and development [43, 44]. In *Arabidopsis*, AtARF8 overexpression results in enhanced *GH3* expression, a 30 % increase of auxin content, and the development of short hypocotyls [35]. These findings indicate that the role of NtARF8 in plant growth is related to the proper level of apical dominance as a function of the auxin signal. At present, we do not have evidence that shows whether this auxin homeostasis mechanism is involved in the NtTTG2-NtARF8 functional pathway for the regulation of tobacco growth and development.

Concomitant changes in the subcellular localization of NtTTG2 and NtARF8 are critical for NtARF8 to execute the function in the transcriptional regulation of the illustrational target gene (Fig. 8). The nuclear localization and transcription regulatory activity of NtARF8 apparently requires the upregulation of the NtARF8 protein. In this case, *NtARF8* gene expression could be induced to ensure further production of the protein that is caused by NtTTG2 overexpression. Thus, nuclear localization of NtTTG2 and NtARF8 likely induces a feedback mechanism involving the transcription of the specific genes. This hypothesized feedback scheme may take place, for example, when NtARF8 executes its function on auxin-responsive genes in the presence of NPR1 to activate immune response genes. In the latter case, NPR1 is hydrolyzed after a single round of gene activation such new NPR1 molecules are used in every round of transcriptional regulation [17]. However, this proteolysis model does not exclude an alternative mechanism, i.e., overexpressed NtTTG2 transports a high amount of NtARF8 into the nucleus, where NtARF8 functions to activate a large number of auxin-responsive genes such as *GH3* (Fig. 8). A central question for both models is how NtARF8 is physically connected with NtTTG2. Because NtTTG2 does not directly interact with NtARF8 (Additional file 8: Figure S7), additional components that fill the apparent gap in NtTTG2-mediated nuclear import of NtARF8 should be identified. The candidates are most likely to be particular members of the impotin protein family as these proteins function as cargo carriers for the nucleocytoplasmic trafficking of proteins and nucleic acids [45]. Unknown mediators such as importins may intercede the linkage between NtTTG2 and NtARF8, and assist NtTTG2 in transporting NtARF8 into the nucleus. Testing of this hypothesis will be the subject of future studies.

## Conclusions

Genetic analyses indicate that ARF8, ARF17, and ARF19 contribute to NtTTG2-regulated tobacco growth and development, whereas ARF8 plays a major role in the process. Molecular evidence indicates that ARF8 is a functional transcriptional activator, whereas the NtTTG2 protein indirectly regulates the function of NtARF8 by modulating its localization to the nucleus. In essence, NtARF8 is an integral component of the NtTTG2 functional pathway that governs the growth and development of tobacco.

## Methods

### Plant growth and treatment

The common tobacco variety, NC89, and transgenic lines in the T3 homozygous generation [4] were used in the present study. Transgenic plants were generated, characterized, and multiplied in this lab, and seeds of WT and transgenic plants were also maintained in this lab. For the experiments performed on roots, seeds were sterilized and germinated and subsequently, seedlings were grown on Murashige and Skoog (MS) agar medium in 10-cm square plates under environment-controlled conditions in a plant growth chamber with a 14-h light (250 μE/m^2^/s at 26 °C) and 10-h dark (23 °C) cycle. Alternatively, seeds were sown and germinated in pots filled with a mixture of sand and potting soil, and subsequently seedlings were grown in the plant growth chamber. At 30 days after seed germination, seedlings were transferred into new pots containing the same substrate and grown under different conditions depending on the experimental purposes. Plants used in the evaluation of vegetative growth were grown in the chamber, and those used for seed production were grown in a greenhouse at 23 °C −26 °C. After continuous growth in the chamber for an additional 20 days, plants were used in the gene silencing experiments or treated for analysis of NAA-induced *NtTTG2* expression. NAA was prepared as a 2 μM aqueous solution amended with 0.02 % (v/v) of surfactant Silwet-77 and applied by spraying over plant tops with an atomizer. A 0.02 % Silwet-77 solution was similarly applied to controls. The treated plants were used in the analysis of gene expression at a 10-h interval in 60 h after treatment.

### Gene expression analysis

Depending on the study purpose, RNA was isolated from intact roots, top 5-cm stems, the top sixth leaves, S3 stage flowers [19], and 15-day-old immature fruits of 70-day-old plants. Northern blots were hybridized to specific probes labeled with digoxigenin [46]. RT-PCR and RT-qPCR were conducted using specific primers (Additional file 9: Table S2) and previously described protocols [47]. The constitutively expressed *EF1α* gene [47, 48] was used as reference. The relative expression level of each tested gene was quantified by RT-qPCR using that of the *EF1α* gene as reference [48].

### Endogenous auxin measurements

Endogenous free auxin (IAA) concentrations were determined by using a previously described protocol [49]. To isolate free IAA, the top sixth leaves of 30-day-old plants, S3 flowers [19], and immature fruits of 70-day-old plants were collected. Approximately 1 g of each organ was ground in liquid nitrogen by using a mortar and pestle. Tissue powders were dissolved in 10 mL of 80 % (v/v) ethanol and supplemented with 0.1 mg of the antioxidant, 2,6-di-tert-butyl-p-cresol. The homogenate was sonicated for 30 min and then centrifuged (4 °C, 10,000 *g*, 10 min). The supernatant was mixed with an equal volume of mineral ether for liquid-phase extraction, which was repeated three times, with the bottom inorganic phase collected each time. The solution from the last extraction was adjusted to pH8.5 and mixed with 0.2 g of inylpyrrolidone, followed by three rounds of liquid-phase extraction using an equal volume of acetic ester and collection of the upper organic phase. IAA in the last organic phase was purified by chromatography using an OASIS MAX Cartridge 3 cc/60 mg hybrid anion exchange resin (Waters Oasis). The solute was analyzed by high-performance liquid chromatography. IAA concentrations in the extracts were determined by using a standard IAA curve method. The levels of free IAA in plant organs were measured and expressed as ng/g fresh tissue.

### VIGS

Partial fragments (207–495 bp) of the *NtARF* genes were cloned by RT-PCR using RNA isolated from leaves and primers that were synthesized according to sequences of the corresponding unigenes (Additional file 9: Table S2). The RT-PCR products were sequenced for confirmation, and every sequence was inserted into a pBinPlus:Y35 DNA-A vector of the TCSV VIGS system [38] to generate various *ARF*-silencing constructs. Every construct was transferred into *Agrobacterium tumefaciens* EHA105 cells [38]. A suspension of recombinant EHA105 cells was infiltrated into the phloem of mid stems of 50-day-old plants that were grown in the chamber. A similar transfection treatment was performed using pure water in the control. Two weeks later, gene silencing efficiencies were analyzed as previously described [10].

### EMSA and ChIP

EMSA and ChIP analyses were conducted by using previously described methods [47]. For EMSA, the ARF8 protein was fused to a His tag for six tandem histidine residues, and a fusion protein was produced by prokaryotic expression [48]. Previously designed WT probe, 5'-CATTATTTACG***TGTCT***GTTTTCCTG-3', and mutant 5'-CATTATTTACG***tagat***GTTTTCCTG -3' [26] were used in the EMSA after labeling with biotin. The chromatin used in the ChIP assay was isolated from the top sixth leaves of 50-day-old plants as described elsewhere [50]. Complexes of chromatin fragments and NtARF8-YFP (ARF8-IP complexes) were precipitated with a specific YFP antibody (Santa Cruz) and the Protein A agarose/salmon sperm DNA beads (Millipore). The NtARF8-IP complexes bound to the beads were collected by centrifugation. Concentrations of the antibody and chromatin were 10 μg and 50 nM per immunoprecipitation, respectively. Similar precipitation and centrifuge procedures were conducted in the absence of the antibody in the control. Chromatin without precipitation were used as input. DNA samples from ChIP, control, and input were analyzed separately by PCR using primers specific to the promoter and CDS of the *GH3* gene (Additional file 9: Table S2). The PCR products were confirmed by sequencing.

### Fluorescence imaging

The *ARF8-YFP* fusion gene was constructed with a plant binary vector pCAMBIA1301 that was transferred into cells of *A. tumefaciens* strain EHA105 by using a previously described method [47]. Roots of 20-day-old plants grown on MS agar medium were transformed by immersion in a suspension of the recombinant EHA105 cells. Approximately 60 h later, root samples were observed under a ZEISS LSM710 confocal microscope. Red and yellow fluorescence were captured at wavelengths of 591–630 and 519–560 nm using argon laser at excitation wavelengths of 561 and 514 nm, respectively [10].

### Immunoblotting

Total proteins were extracted from the top sixth leaves [51], the cytoplasmic fraction, and the nuclear fraction [52] of 50-day-old plants grown in the chamber. Western blots were hybridized with the specific antibody against YFP (Novagen) or the specific NtTTG2 antibody, which was produced by immunizing a New Zealand white rabbit in our previous study [47]. PEPC and histone H3 that were used as cytoplasmic and nuclear markers [4, 41] were hybridized with a specific PEPC antibody (Rockland) and histone H3 antibody (Abcam), respectively. Hybridized blots were probed by using a horseradish peroxidase-conjugated secondary antibody (Beyotime) according to the manufacturer’s recommendations.

### Statistical analysis

All experiments were conducted for at least three times with similar results. Quantitative data were analyzed by using the commercial IBM SPSS19.0 software package [53]. Homogeneity-of-variance in data was determined by using the Levene test, and the formal distribution pattern of the data was confirmed by using the Kolmogorov-Smirnov test and P-P plots. Data were subjected to ANOVA, along with Fisher’s least significant difference test and Tukey-Kramer’s test, respectively. Significance was tested for differences in multiple comparisons of various plant genotypes.

## Abbreviations

ARF, AUXIN RESPONSIVE FACTOR; *AUX/IAA*, Auxin/Indole-Acetic Acid inducible; ChIP, Chromatin Immunoprecipitation; EMSA, Electrophoresis mobility shift assay; *GH3*, *Gretchen Hagen 3*; NAA, 1-naphthaleneacetic acid; NPR1, NONEXPRESSER OF PATHOGENESIS-RELATED GENES1; ParA1, An elicitin protein that is produced by an oomycete pathogen; PEPC, Phosphoenolpyruvate carboxylase; RFP, Red-fluorescent protein; RT-PCR, Reverse transcriptase-polymerase chain reaction; RT-qPCR, Quantitative real-time RT-PCR; *SAUR*, *Small Auxin Up RNA*; TCSV, *Begomovirus* sp. *tobacco curly shoot virus*; TTG, TRANSPARENT TESTA GLABRA; VIGS, Virus-induced gene silencing; YFP, Yellow-fluorescent protein

## Additional files

Additional file 1: Figure S1. IAA concentrations in leaves, flowers, and fruits of *NtTTG2*-related tobacco genotypes. (PDF 45 kb) Additional file 2: Table S1. List of newly specified NtTTG2-regulated tobacco *ARF* genes (PDF 18 kb) Additional file 3: Figure S2. The chronological course of foliar *NtARF8*, *NtARF17*, and *NtARF19* expression during the vegetative growth process. (PDF 40 kb) Additional file 4: Figure S3.
*NtARF8* expression in different organs of *NtTTG2*-related tobacco genotypes. (PDF 50 kb) Additional file 5: Figure S4. The effects of *NtARF8* and *NtTTG2* overexpression on plant growth. (PDF 84 kb) Additional file 6: Figure S5. Alignments of selected *ARF8* homologs. (PDF 342 kb) Additional file 7: Figure S6. Foliar expression levels of *NtTTG2*, *NtARF8*, and *GH3* under backgrounds of single and concurrent *NtTTG2* and *NtARF8* silencing or overexpression. (PDF 68 kb) Additional file 8: Figure S7. Yeast two-hybrid and bimolecular fluorescence complementation analyses of NtTTG2 and NtARF8. (PDF 78 kb) Additional file 9: Table S2. List of genes tested and primers used in this study. (PDF 48 kb)
