# Supplementary material for: Tobacco TTG2 regulates vegetative growth and seed production via the predominant role of ARF8 in cooperation with ARF17 and ARF19
Source: BMC Plant Biol. 2016 Jun 2;16:126. doi: 10.1186/s12870-016-0815-3 (PMC4890496; doi:10.1186/s12870-016-0815-3)
Supplement: Additional file 1: Figure S1. — IAA concentrations in leaves, flowers, and fruits of NtTTG2-related tobacco genotypes. (PDF 45 kb) [file 12870_2016_815_MOESM1_ESM.pdf]

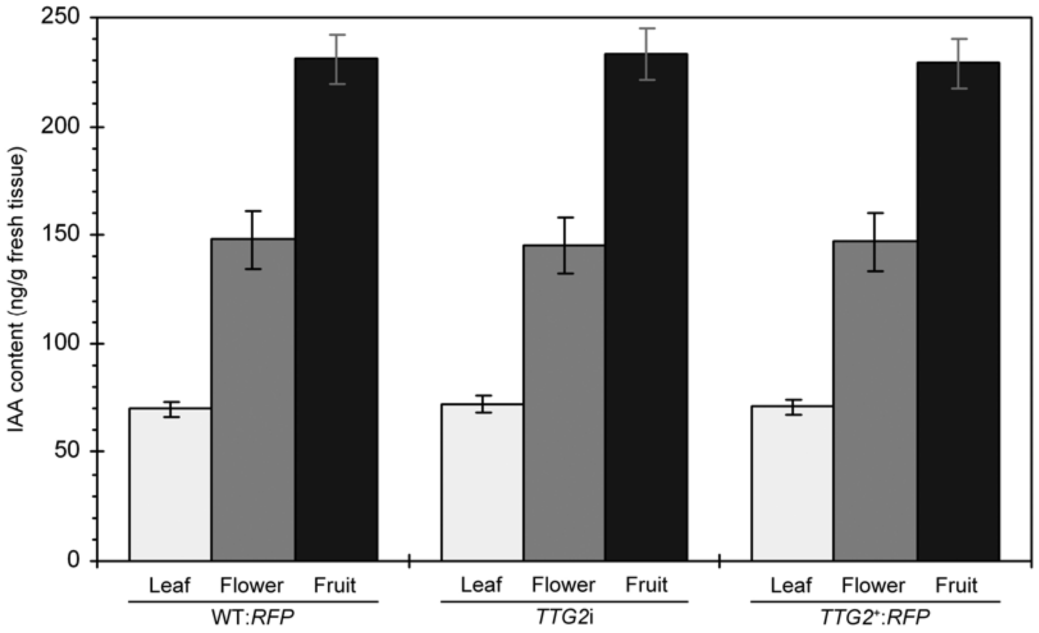

Additional File 1: Figure S1 IAA concentrations in leaves, flowers, and fruits of *NtTTG2*-related tobacco genotypes. IAA concentrations were determined with the top sixth leaves of 30-day-old plants, S3 flowers, and immature fruits from 70-day-old plants grown in the green house. Data shown are mean values ± SEM bars (*n* = 3 experimental replicates).
