# Supplementary material for: Tobacco TTG2 regulates vegetative growth and seed production via the predominant role of ARF8 in cooperation with ARF17 and ARF19
Source: BMC Plant Biol. 2016 Jun 2;16:126. doi: 10.1186/s12870-016-0815-3 (PMC4890496; doi:10.1186/s12870-016-0815-3)
Supplement: Additional file 2: Table S1. — List of newly specified NtTTG2-regulated tobacco ARF genes (PDF 18 kb) [file 12870_2016_815_MOESM2_ESM.pdf]

Additional File 2: Table S1 List of newly specified NtTTG2-regulated tobacco *ARF* genes

Nicotiana tabacum ARF gene name	Unigene code[a]	Most similar orthologs[b] (GenBank accession number)
NtARF1	comp31238_c0	*Nicotiana benthamiana ARF1* (GQ859156)
NtARF2	comp31531_c0	*Arabidopsis thaliana ARF2* (AY669787); *N. tabacum ARF2-like* (DQ340258)
NtARF5	comp19484_c2	*Solanum lycopersicum ARF5* (NM_001247616); *A. thaliana ARF5* (GU348464)
NtARF6L	comp40625_c0	*N. tomentosiformis ARF6-like* (XM_009603918)
NtARF8	comp42904_c0	*S. lycopersicum ARF8-1* (XM_010319347)
	comp30272_c0	*N. sylvestris ARF8-like* (XM_009771234); *A. thaliana ARF8* (EU550096)
NtARF9	comp41729_c1	*Glycine max ARF9* (KR820243); *A. thaliana ARF9* (GU348484)
NtARF11	comp38086_c0	*A. thaliana ARF11* (AY669791)
NtARF16	comp1238_c0	*S. lycopersicum ARF16* (NM_001247951)
NtARF17	comp39443_c0	*S. lycopersicum ARF17* (XM_010314139); *A. thaliana ARF17* (EU550167)
NtARF18	comp41729_c2	*Vitis vinifera ARF18* (CP002686)*; A. thaliana ARF18* (GU348513)
NtARF19	comp38146_c2	*S. lycopersicum ARF19* (NM_001247811); *A. thaliana ARF19* (AY669794)
NtARF19L	comp26539_c0	*N. tomentosiformis ARF19*-like (XM_009596245.1)

[a] The tobacco transcriptome data are available at http://www.ncbi.nlm.nih.gov/sra/?term=SRX363387.

[b] Tobacco *Nicotiana* species are considered in the first priority over other plant species.
