# Supplementary material for: Tobacco TTG2 regulates vegetative growth and seed production via the predominant role of ARF8 in cooperation with ARF17 and ARF19
Source: BMC Plant Biol. 2016 Jun 2;16:126. doi: 10.1186/s12870-016-0815-3 (PMC4890496; doi:10.1186/s12870-016-0815-3)
Supplement: Additional file 3: Figure S2. — The chronological course of foliar NtARF8, NtARF17, and NtARF19 expression during the vegetative growth process. (PDF 40 kb) [file 12870_2016_815_MOESM3_ESM.pdf]

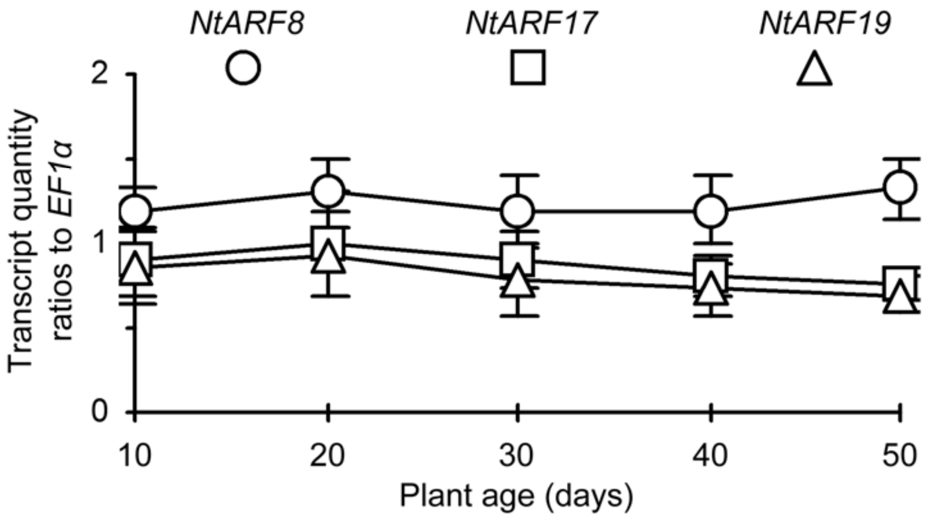

Additional File 3: Figure S2 The chronological course of foliar *NtARF8*, *NtARF17*, and *NtARF19* expression during the vegetative growth process. Gene expression in the top sixth leaves of wild-type (WT) plants grown in the chamber was quantified by RT-qPCR. Data shown are mean values ± SEM bars (*n* = 3 experimental replicates).
