# Supplementary material for: Tobacco TTG2 regulates vegetative growth and seed production via the predominant role of ARF8 in cooperation with ARF17 and ARF19
Source: BMC Plant Biol. 2016 Jun 2;16:126. doi: 10.1186/s12870-016-0815-3 (PMC4890496; doi:10.1186/s12870-016-0815-3)
Supplement: Additional file 4: Figure S3. — NtARF8 expression in different organs of NtTTG2-related tobacco genotypes. (PDF 50 kb) [file 12870_2016_815_MOESM4_ESM.pdf]

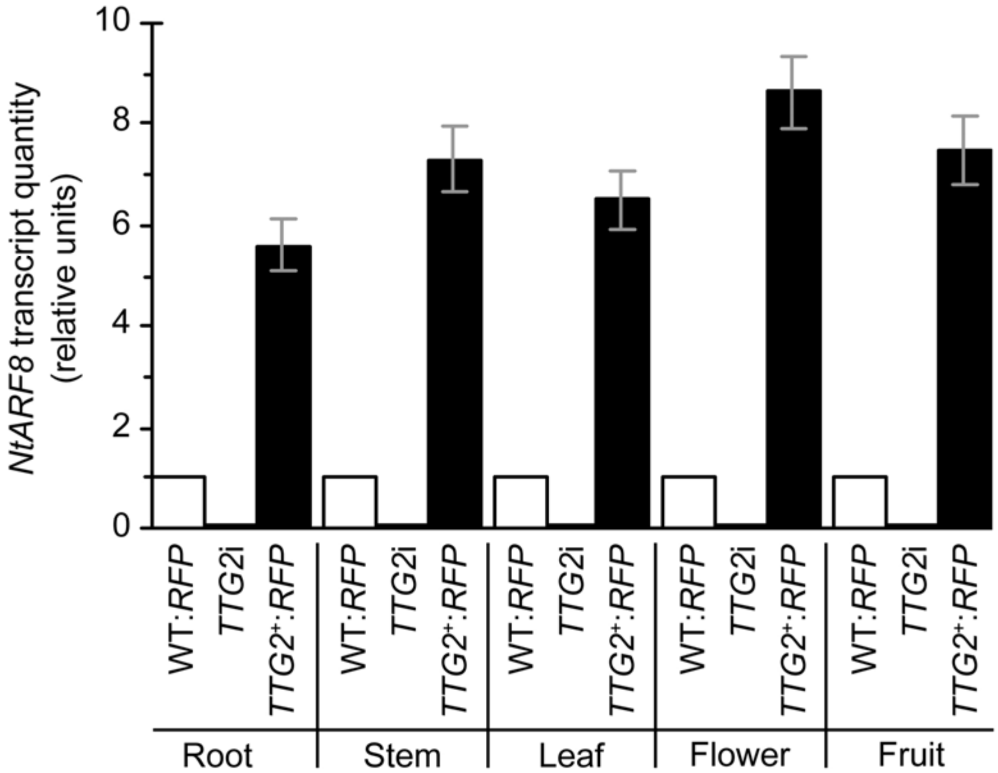

Additional File 4: Figure S3 *NtARF8* expression in different organs of *NtTTG2*-related tobacco genotypes. The RT-qPCR protocol was used to quantify gene expression levels in intact roots, stems, and the top sixth leaves of 30-day-old plants, and in S3 flowers and immature fruits of 70-day-old plants grown in the green house. Relative levels of gene expression are setup as 1 in WT:*RFP* organs to highlight the effects of gene silencing and overexpression. Data shown are mean values ± SEM bars (n = 3 experimental replicates).
