# Supplementary material for: Tobacco TTG2 regulates vegetative growth and seed production via the predominant role of ARF8 in cooperation with ARF17 and ARF19
Source: BMC Plant Biol. 2016 Jun 2;16:126. doi: 10.1186/s12870-016-0815-3 (PMC4890496; doi:10.1186/s12870-016-0815-3)
Supplement: Additional file 5: Figure S4. — The effects of NtARF8 and NtTTG2 overexpression on plant growth. (PDF 84 kb) [file 12870_2016_815_MOESM5_ESM.pdf]

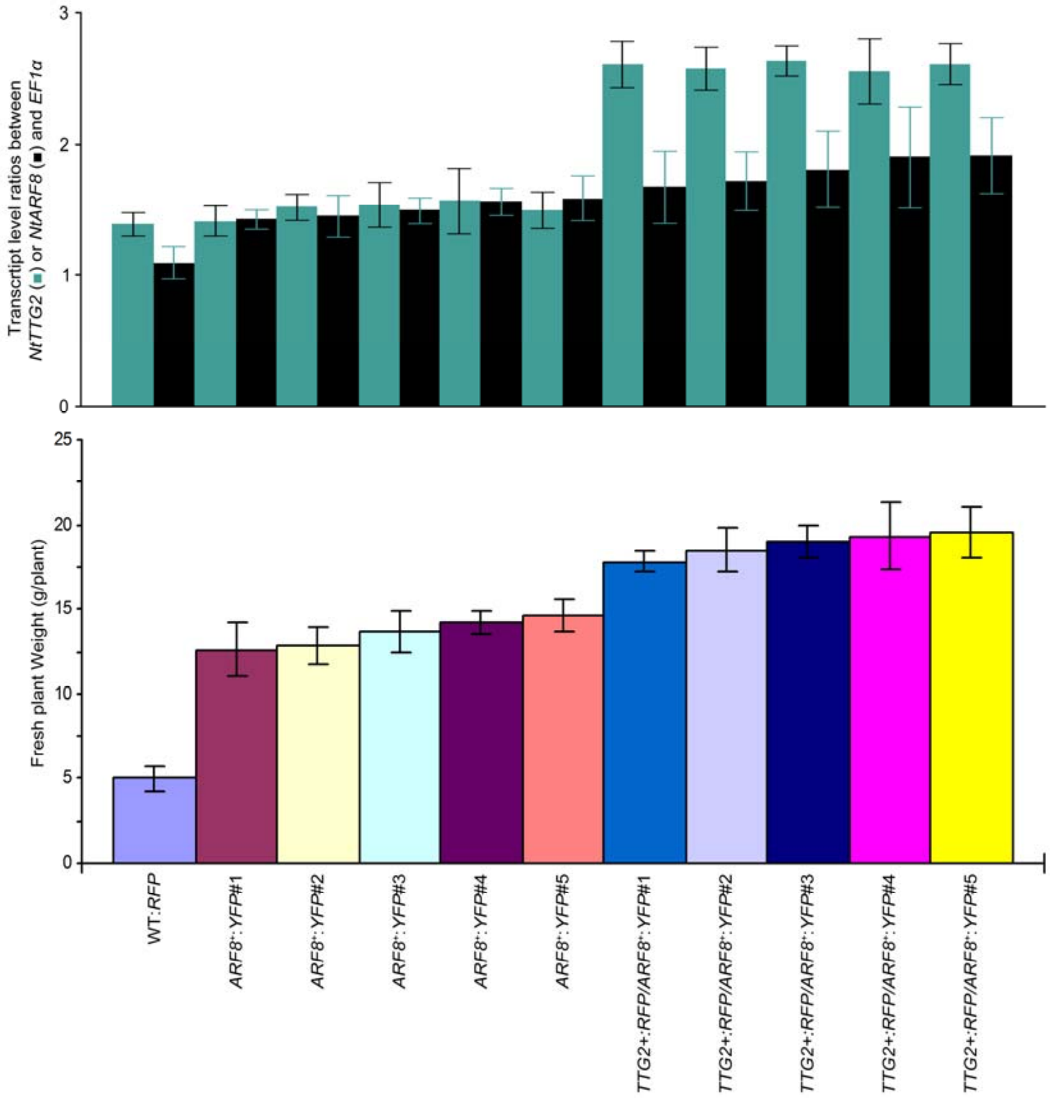

Additional File 5: Figure S4 The effects of *NtARF8* and *NtTTG2* overexpression on plant growth. Gene expression analyses were performed on the top sixth leaves of 30-day-old plants and fresh weight of 50-day-old plants was determined. The bar graphs represent means ± SEMs of replicate results (*n* = 3 experimental replicates).
