# Supplementary material for: Tobacco TTG2 regulates vegetative growth and seed production via the predominant role of ARF8 in cooperation with ARF17 and ARF19
Source: BMC Plant Biol. 2016 Jun 2;16:126. doi: 10.1186/s12870-016-0815-3 (PMC4890496; doi:10.1186/s12870-016-0815-3)
Supplement: Additional file 6: Figure S5. — Alignments of selected ARF8 homologs. (PDF 342 kb) [file 12870_2016_815_MOESM6_ESM.pdf]

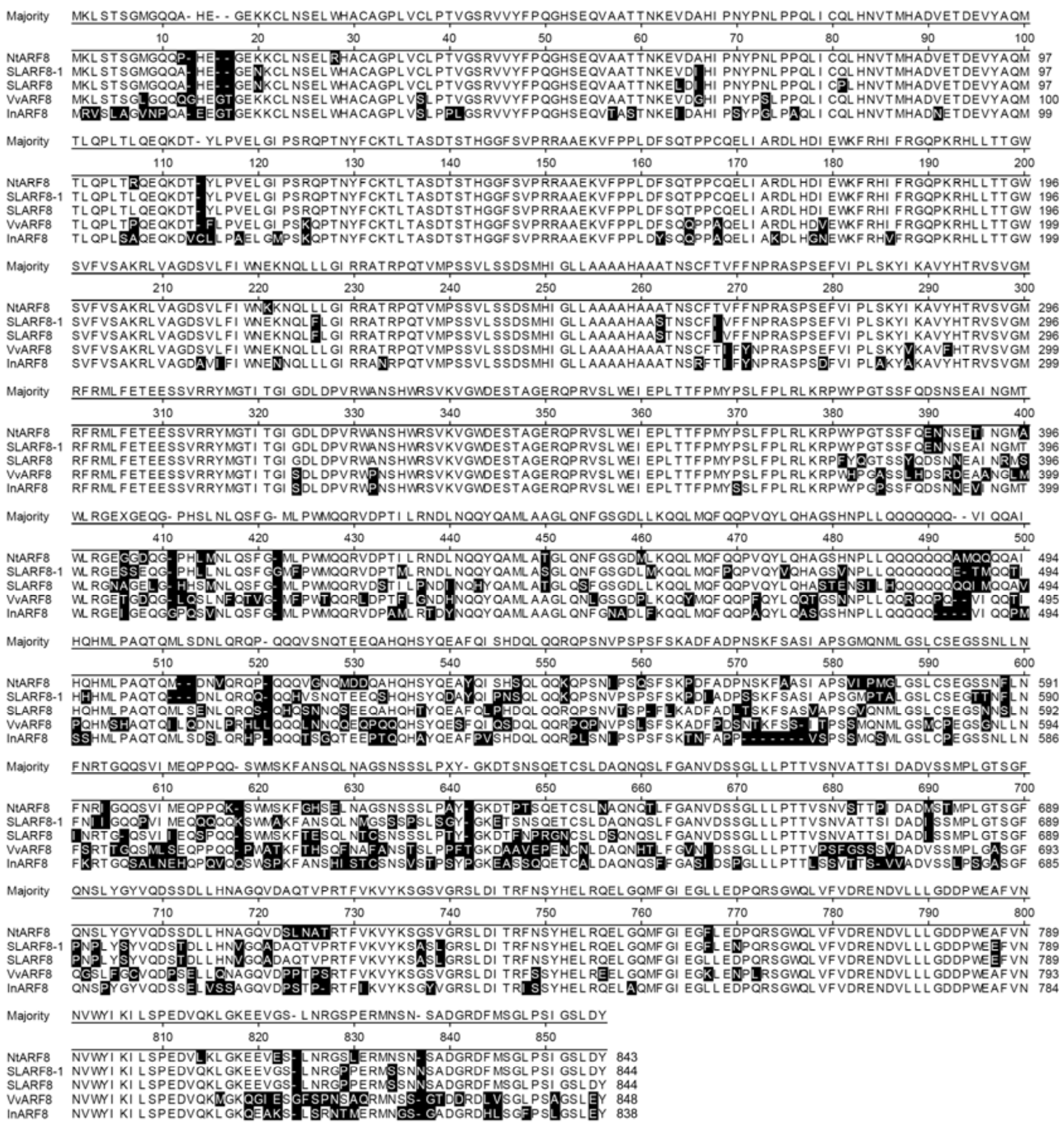

Additional File 6: Figure S5 Alignments of selected *ARF8* homologs. The sequence of NtARF8 was aligned with homologs from tomato (*Solanum lycopersicum*; accession numbers HM560979 and EF667342.18), common grape vine (*Vitis vinifera*; XP002266678.2), and morning glory (*Ipomoea nil*; EF216864). Different residues are shaded.
