# Supplementary material for: Tobacco TTG2 regulates vegetative growth and seed production via the predominant role of ARF8 in cooperation with ARF17 and ARF19
Source: BMC Plant Biol. 2016 Jun 2;16:126. doi: 10.1186/s12870-016-0815-3 (PMC4890496; doi:10.1186/s12870-016-0815-3)
Supplement: Additional file 7: Figure S6. — Foliar expression levels of NtTTG2, NtARF8, and GH3 under backgrounds of single and concurrent NtTTG2 and NtARF8 silencing or overexpression. (PDF 68 kb) [file 12870_2016_815_MOESM7_ESM.pdf]

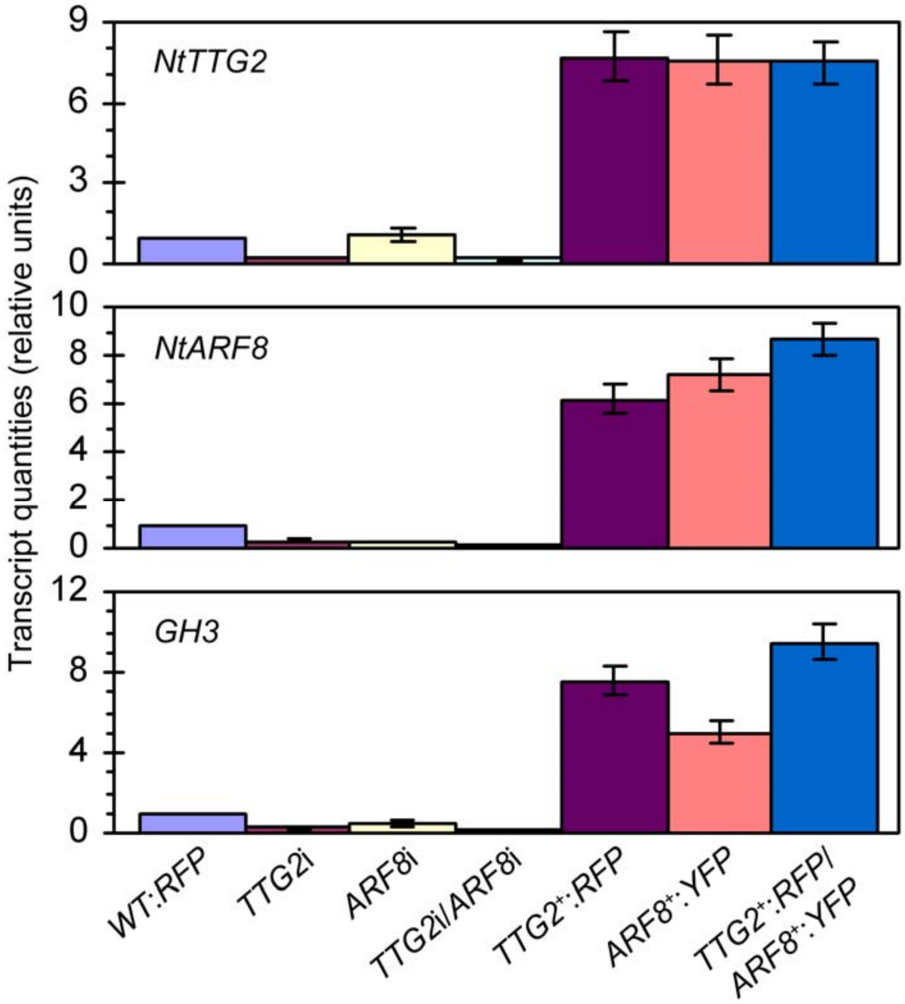

Additional File 7: Figure S6 Foliar expression levels of *NtTTG2*, *NtARF8*, and *GH3* under backgrounds of single and concurrent *NtTTG2* and *NtARF8* silencing or overexpression. The RT-qPCR protocol was used to quantify gene expression levels in the top sixth leaves of 30-day-old plants. Data shown are mean values ± SEM bars (*n* = 3 experimental replicates).
