# Supplementary material for: Tobacco TTG2 regulates vegetative growth and seed production via the predominant role of ARF8 in cooperation with ARF17 and ARF19
Source: BMC Plant Biol. 2016 Jun 2;16:126. doi: 10.1186/s12870-016-0815-3 (PMC4890496; doi:10.1186/s12870-016-0815-3)
Supplement: Additional file 8: Figure S7. — Yeast two-hybrid and bimolecular fluorescence complementation analyses of NtTTG2 and NtARF8. (PDF 78 kb) [file 12870_2016_815_MOESM8_ESM.pdf]

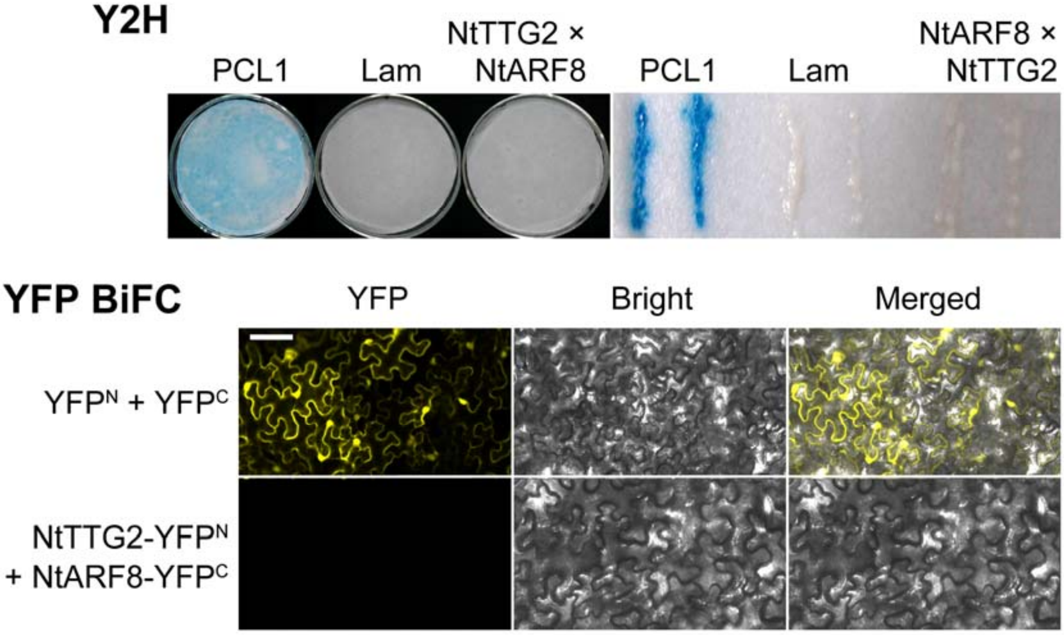

Additional File 8: Figure S7 Yeast two-hybrid and bimolecular fluorescence complementation analyses of NtTTG2 and NtARF8. The Yeast two-hybrid assay for the protein combinations shown as bait × prey in comparison with positive control (PCL1) and negative control (Lam). The bimolecular fluorescence complementation assay of NtTTG2 and NtARF8 using yellow-fluorescent protein (YFP) as a probe. NtTTG2 fused to the N-terminal region of YFP (Y^N) was combined with NtARF8 ligated to the C-terminal region (Y^C) in the assay. This combination did not show interacting signal in contrast to YFP used as a positive control.
