# Supplementary material for: Tobacco TTG2 regulates vegetative growth and seed production via the predominant role of ARF8 in cooperation with ARF17 and ARF19
Source: BMC Plant Biol. 2016 Jun 2;16:126. doi: 10.1186/s12870-016-0815-3 (PMC4890496; doi:10.1186/s12870-016-0815-3)
Supplement: Additional file 9: Table S2. — List of genes tested and primers used in this study. (PDF 48 kb) [file 12870_2016_815_MOESM9_ESM.pdf]

Additional File 9: Table S2 List of genes tested and primers used in this study

Gene	Primers / product size (bp)
ARF1	5'-CGC**GGATCC**AATGGCGTCGGATGTGATG-3' (***Bam*H I**),<br>5'-GC***TCTAGA***AGGTAGTTCCACAGGCTTATCAAT-3' (***Xba* I**) / 303
ARF2	5'-CGC**GGATCC**CGCACTTACAAACCTACTCC-3',<br>5'-GC***TCTAGA***TGAGGCCAATACAGATGAAG-3' / 396
ARF5	5'-CGC**GGATCC**AGCCATAACCAAAGCGACAT-3',<br>5'-GC***TCTAGA***CTTGCTGCCATGAGCTATTC-3' / 319
ARF6L	5'-CG**GGATCC**GGTGGTATTTAGTTGTGGAAGTGGG-3',<br>5'-GC***TCTAGA***GAAGCTGACAAATTAGCTGAGGTGG-3' / 448
ARF8 (comp42904_c0)*	5'-CG**GGATCC**TTCAACATCAGGAATGGGTCAGC-3',<br>5'-GC***TCTAGA***GGCAAGTTCGGGTAATTGGGTAT-3 / 207
ARF8 (comp30272_c0)*	5'-CGC**GGATCC**TTGGTGGGAGATTTTAGGGTTTG-3',<br>5'-GC***TCTAGA***AGTAGGCTGCTTGCTAGGGAT-3' / 420
ARF9	5'-CGC**GGATCC**GGCTG GAGTTCTGTAAGATGGTC-3',<br>5'-GC***TCTAGA***CACAAAGGTATTCACAATTTCCAG-3' / 416
ARF11	5'-CGC**GGATCC**AGCCTAAGTGTTGCCGTTGT-3',<br>5'-GC***TCTAGA***AAGTCCATTGGGAGGGTTAT-3' / 364
ARF16	5'-CG**GGATCC**TGAAGAAACCGCCAAGGTGAATGAG-3',<br>5'-GC***TCTAGA***GGTGGTATTGGTGGGATGGAACTGA-3' / 335
ARF17	5'-CGC**GGATCC**CCTTGGGTTGGGCACTGAC-3',<br>5'-GC***TCTAGA***CTGAGGCTCTTTGGTGTTGG-3' / 460
ARF18	5'-CG**GGATCC**ACCTCATCCTGAAACGCATACCTAC-3',<br>5'-GC***TCTAGA***CCAACCTCGAAGACACCTGCTTAC-3' / 341
ARF19	5'-CGC**GGATCC**TTTGGGAGATTGAACCTGTA-3',<br>5'-GC***TCTAGA***CATGTTCATCCATTGGACTAAG-3' / 215
ARF19L	5'-CG**GGATCC**CCCTTCCAACACGAGCTAACCCTCTA-3',<br>5'-GC***TCTAGA***TCTCCGCAAGAAGTCCAGCAAATGA-3' / 365
EF1α (KF286542.1)	5'-AGACCACCAAGTACTACTGCAC-3',<br>5'-CCACCAATCTTGTACACATCC-3' / 495
GH3 CDS (AF123503)	5'-TAACCTTCCACTCTTAGCCAATG-3',<br>5'-GCCAGCCACTTTCAGATAC-3' / 212
GH3 promoter (Heinekamp et al., 2004)	5'-CTCGTAGGAGGTCATGTAGCATG-3',<br>5'-GTTTGTGTTGGAATTAGATAACGAAGG-3' / 458

*Sequence data are available at http://www.ncbi.nlm.nih.gov/sra/?term=SRX363387.
